# Palmitoylation of PKCδ by ZDHHC5 in hypothalamic microglia presents as a therapeutic target for fatty liver disease

**DOI:** 10.7150/thno.89602

**Published:** 2024-01-01

**Authors:** Yan-Hang Wang, Xin Chen, Yi-Zhen Bai, Peng Gao, Zhuo Yang, Qiang Guo, Ying-Yuan Lu, Jiao Zheng, Dan Liu, Jun Yang, Peng-Fei Tu, Ke-Wu Zeng

**Affiliations:** 1 State Key Laboratory of Natural and Biomimetic Drugs, School of Pharmaceutical Sciences, Peking University, Beijing 100191, China.; 2 Department of Neurosurgery, Peking University Third Hospital, Beijing 100191, China.; 3 School of Chinese Materia Medica, Beijing University of Chinese Medicine, Beijing 100102, China.; 4 Proteomics Laboratory, Medical and Healthy Analytical Center, Peking University Health Science Center, Beijing 100191, China.

**Keywords:** hepatic lipid metabolism, protein kinase Cδ (PKCδ), palmitoylation modification, hypothalamic microglia, artemether

## Abstract

The hypothalamus plays a fundamental role in controlling lipid metabolism through neuroendocrine signals. However, there are currently no available drug targets in the hypothalamus that can effectively improve human lipid metabolism. In this study, we found that the antimalarial drug artemether (ART) significantly improved lipid metabolism by specifically inhibiting microglial activation in the hypothalamus of high-fat diet-induced mice. Mechanically, ART protects the thyrotropin-releasing hormone (TRH) neurons surrounding microglial cells from inflammatory damage and promotes the release of TRH into the peripheral circulation. As a result, TRH stimulates the synthesis of thyroid hormone (TH), leading to a significant improvement in hepatic lipid disorders. Subsequently, we employed a biotin-labeled ART chemical probe to identify the direct cellular target in microglial cells as protein kinase Cδ (PKCδ). Importantly, ART directly targeted PKCδ to inhibit its palmitoylation modification by blocking the binding of zinc finger DHHC-type palmitoyltransferase 5 (ZDHHC5), which resulted in the inhibition of downstream neuroinflammation signaling. In vivo, hypothalamic microglia-specific PKCδ knockdown markedly impaired ART-dependent neuroendocrine regulation and lipid metabolism improvement in mice. Furthermore, single-cell transcriptomics analysis in human brain tissues revealed that the level of PKCδ in microglia positively correlated with individuals who had hyperlipemia, thereby highlighting a clinical translational value. Collectively, these data suggest that the palmitoylation of microglial PKCδ in the hypothalamus plays a role in modulating peripheral lipid metabolism through hypothalamus-liver communication, and provides a promising therapeutic target for fatty liver diseases.

## Introduction

Lipid metabolism plays a critical role in fatty liver disease 1. Currently, the strategies employed to prevent and manage hepatic lipid metabolism disorders primarily revolve around targeting canonical molecular markers including 3-hydroxy-3-methyl glutaryl coenzyme A (HMG-CoA) reductase, peroxidase proliferative activity receptor, proprotein convertase subtilisin/kexin 9 and lipase 2. However, we still need to explore novel targets that differs from existing mechanisms to overcome potential adverse reactions, such as elevated transaminase levels, rhabdomyolysis, gastrointestinal symptoms, and rash 3-6.

Increasing evidence suggests that the neuroendocrine system plays a crucial role in the regulation of lipid metabolism. Clinical studies have demonstrated that individuals with low levels of thyroid hormone, a significant neuroendocrine messenger, exhibit elevated expression of various mitochondrial enzymes involved in β-oxidation and oxidative phosphorylation. These enzymes include carnitine palmitoyltransferase 1A (CPT1A), mitochondrial uncoupling protein 2 (UCP2), and pyruvate dehydrogenase kinase isoform 4 (PDK4) 7-9. Consequently, these individuals are more likely to experience a disruption in lipid metabolism. Moreover, decreased thyroid hormone level (thyroxine and triiodothyronine) or impaired hypothyroidism may lead to serious hepatic lipid accumulation and lipid metabolites increase, indicating a potential correlation between neuroendocrine signal and fatty liver disease 10. Furthermore, as a synthetic thyroid hormone, *l*-thyroxine (*l*-THY) has been recently found to improve hepatic lipid metabolism and reduce body fat percentage in obese patients 11. Therefore, effective control of neuroendocrine signal especially thyroid hormone level may represent a promising direction for treating lipid metabolism disorders.

Hypothalamus is a critical brain region that connects the neuroendocrine system to peripheral physiological functions 12. In the paraventricular (PVN) nucleus of hypothalamus, TRH neurons secrete TRH, thereby stimulating the production of pituitary thyrotropin (also thyroid-stimulating hormone, TSH) for thyroid hormone biosynthesis, including total thyroxine (T4) and total triiodothyronine (T3) 13-14. Thus, pathological hypothalamus damage such as microglia-mediated neuroinflammation can result in dysfunction of neuroendocrine to cause lipid metabolism disorder and obesity 15. In particular, recent studies in molecular mechanism support direct association of high lipid exposure with microglial activation via evoking multiple neuroinflammatory signals including Toll-like receptor, NLR family pyrin domain containing 3 (NLRP3) inflammasome and nuclear factor-κB (NF-κB) cascades 16-18. Hence, these studies provide evidence that neuroinflammation in the hypothalamus, mediated by microglia, is implicated in modulating neuroendocrine signaling to influence peripheral lipid metabolism.

Extensive research has consistently highlighted the remarkable biological potential of artemisinin-related compounds in various biological processes, including the mitigation of hepatic fibrosis, inhibition of hepatitis, and facilitation of glucose metabolism 19-20. These compelling findings strongly suggest that these compounds may also possess the capacity to augment lipid metabolism. In this study, we discovered that ART, a major derivative of antimalarial drug artemisinin, significantly improved hepatic lipid disorders upon high-fat diet (HFD) exposure via drug repositioning. Unexpectedly, ART did not directly regulate lipid accumulation in hepatocytes, but specifically inhibited microglia-mediated neuroinflammation in the hypothalamus to protect adjacent TRH neurons, thereby regulating neuroendocrine for hepatic lipid disorder improvement. Particularly, ART inhibited PKCδ palmitoylation by blocking the palmitoyltransferase ZDHHC5 binding, which resulted in neuroinflammation suppression and lipid metabolism improvement. PKCδ-specific knockdown in hypothalamic microglia also significantly reversed ART-dependent hepatic lipid metabolism improvement in mice. Furthermore, single-cell transcriptomic analysis of clinical brain tissue also observed a previously unappreciated association of PKCδ level in active microglia with dyslipidemia patients.

Collectively, microglial PKCδ in hypothalamus holds promise as a potential therapeutic target for enhancing lipid metabolism, particularly in fatty liver diseases, which differs significantly from current concepts of lipid metabolism regulation.

## Results

### Drug Repositioning Identifies Antimalarial Drug ART as a Lipid-Lowering Agent

**Figure 1:**
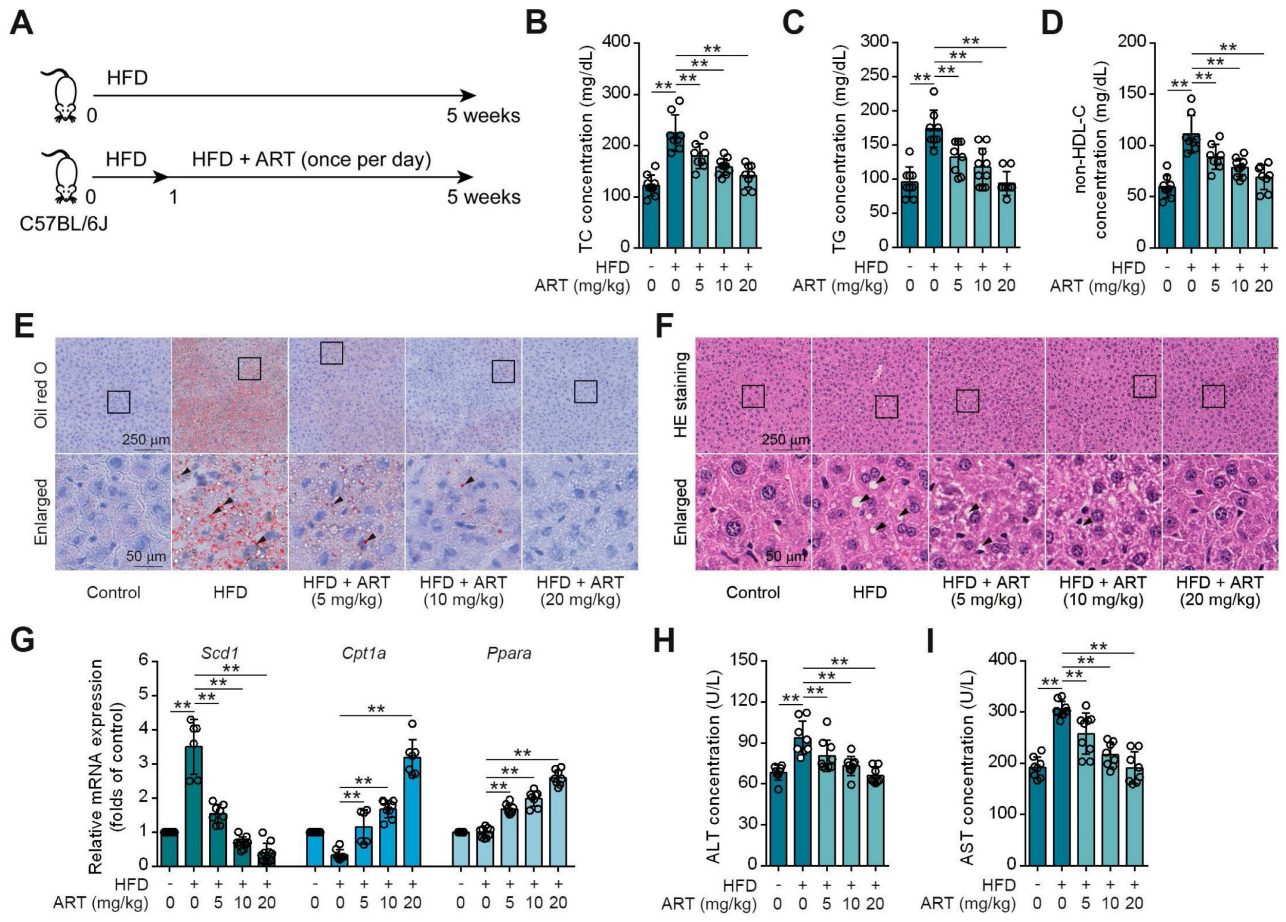
ART inhibits HFD-fed hepatic lipid metabolism disorder in C57BL/6J mice. (A) Experimental design of ART treatment in HFD-fed C57BL/6J mice. (B-D) TC (B), TG (C), and non-HDL-C (D) assay of HFD-fed C57BL/6J mice treated with ART in serum (n = 7-10 mice each group). (E) Oil red O staining of hepatic lipid accumulation in HFD-fed C57BL/6J mice treated with ART. (F) HE staining of hepatic lipid damage in HFD-fed C57BL/6J mice treated with ART. (G) *Scd1*, *Cpt1a* and *Ppara* mRNA levels of HFD-fed C57BL/6J mice treated with ART (n = 6-10 mice each group). (H-I) ALT (H), and AST (I) assay of HFD-fed C57BL/6J mice treated with ART in serum (n = 7-9 mice each group). Statistical comparisons were analyzed by one-way ANOVA analysis followed by Student's t-test. Data are presented as mean ± SD. ^**^p < 0.01.

Drug repositioning is a promising strategy to develop novel treatments for metabolic diseases, thus improving traditional drug development 21. HFD-fed mice were utilized as an experimental model to investigate lipid metabolism, specifically focusing on lipid metabolism disorders 22. To explore whether antimalarial drug ART attenuated HFD-induced lipid metabolism disorder, C57BL/6J mice were fed either a control diet or a HFD for 5 weeks with ART treatment (Figure 1A). As shown in Figure 1B-1D, HFD treatment significantly promoted serum total cholesterin (TC), total glyceride (TG) and non-high-density lipoprotein-cholesterin (non-HDL-C) levels, which were suppressed by ART in a dose-dependent manner. Moreover, oil red O staining showed that HFD treatment promoted lipid droplet accumulation in liver, which was markedly reversed by ART (Figure 1E and S1A). H&E staining also showed that ART ameliorated lipid metabolism disorder-associated steatosis (Figure 1F and S1B). Further, quantitative real time-polymerase chain reaction (RT-PCR) analysis demonstrated a significant increase in the expression of lipid-metabolism-related genes, namely *carnitine palmitoyltransferase 1A* (*Cpt1a*) and *peroxisome proliferator activated receptor alpha* (*Ppara*) in the livers of subjects receiving ART treatment. Additionally, the expression of the lipid synthesis gene, *stearoyl-coenzyme A desaturase 1* (*Scd1*), which is upregulated by HFD treatment, was observed to be down-regulated following oral administration of ART (Figure 1G). A similar pattern was observed in the case of lipid-metabolism-related proteins. The administration of ART significantly augmented the expressions of CPT1A and PPARA proteins, while concomitantly suppressing the expression of SCD1 protein (Figure S1C). Meanwhile, we found that ART obviously decreased serum glutamic-pyruvic transaminase (ALT) and glutamic-oxaloacetic transaminase (AST) levels, and normalized hepatic superoxide dismutase (SOD), glutathione peroxidase (GSH-PX) activities and malondialdehyde (MDA) level, confirming an obvious hepatoprotective effect (Figure 1H-1I, S1D-S1F). To verify the effect of ART against lipid metabolism disorder, we used another Balb/c mouse strain to test our previous observations 23. Here, we found ART showed similar improvement effects on lipid metabolism, including blood lipid levels (Figure S1G-S1I), hepatic lipid accumulation (Figure S1J-S1K), lipid metabolism-related gene expressions, protein expressions and oxidative stress (Figure S1L-S1R). Taken together, these results indicate that ART serves as a potent lipid-lowering agent that improves hepatic lipid metabolism disorder.

### ART Improves Hepatic Lipid Disorder through Activating Neuroendocrine System

To investigate how ART regulated lipid metabolism, we generated the palmitic acid (PA) or oleic acid (OA)-induced human hepatocytes (LO2) model. Nile red staining showed that PA or OA treatment obviously promoted lipid accumulation in LO2 cells, but ART did not prevent these changes (Figure S2A-S2B). Similar observation showed that ART did not block lipid accumulation in PA or OA-induced human HepG2 cells (Figure S2C-S2D). Besides, ART did not show marked effect on *Cpt1a*, and *Ppara* gene expressions in PA-induced LO2 cells (Figure S2E-S2F). In order to gain further insight into the influence of ART on hepatic lipid metabolism, we conducted experiments using primary hepatocytes. The results obtained from our investigation revealed that ART did not enhance the expression of *Cpt1a* and *Ppara* genes or proteins when subjected to PA or OA treatment (Figure S2G-S2L). Thus, these results indicate that ART dose not directly regulate hepatic lipid metabolism.

Previous studies have demonstrated that small intestinal enterocyte uptake and absorption are associated with the development of lipid metabolism disorder 24. To clarify whether ART affected small intestinal enterocyte uptake, Caco-2 transport experiments were carried out. We found that stearic acid (SA), PA, OA, and cholesterol (CHO) were transported from the apical (AP) to the basolateral (BL) side, which was not affected by ART (Figure S3A-S3D). These results indicate that ART dose not interfere with the intestinal absorption. Besides, ART showed no obvious effect on body weight and food intake (Figure S3E-S3F).

**Figure 2:**
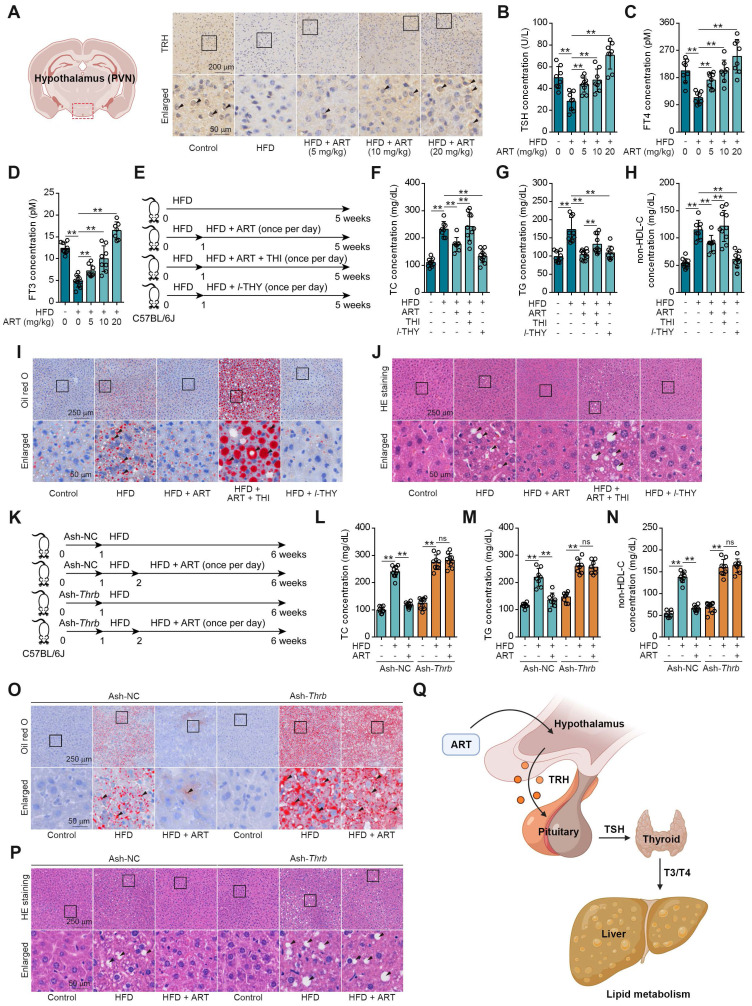
TH mediates ART treatment on HFD-fed hepatic lipid metabolism disorder in C57BL/6J mice. (A) TRH immunohistochemical staining of PVN region in HFD-fed C57BL/6J mice treated with ART. (B-D) TSH (B), FT4 (C), and FT3 (D) assay of HFD-fed C57BL/6J mice treated with ART in serum (n = 7-10 mice each group). (E) Experimental design of ART, THI or *l*-THY treatment in HFD-fed C57BL/6J mice. (F-H) TC (F), TG (G), and non-HDL-C (H) assay of HFD-fed C57BL/6J mice treated with ART (20 mg/kg), THI (15 mg/kg) or *l*-THY (1 mg/kg) in serum (n = 8-10 mice each group). (I) Oil red O staining of hepatic lipid accumulation in HFD-fed C57BL/6J mice treated with ART (20 mg/kg), THI (15 mg/kg) or *l*-THY (1 mg/kg). (J) HE staining of hepatic lipid damage in HFD-fed C57BL/6J mice treated with ART (20 mg/kg), THI (15 mg/kg) or *l*-THY (1 mg/kg). (K) Experimental design of ART treatment in Ash-*Thrb*-injected C57BL/6J mice fed on HFD. (L-N) TC (L), TG (M), and non-HDL-C (N) assay of Ash-*Thrb*-injected C57BL/6J mice treated with HFD or ART (20 mg/kg) in serum (n = 8-10 mice each group). (O) Oil red O staining of hepatic lipid accumulation in Ash-*Thrb*-injected C57BL/6J mice treated with HFD or ART (20 mg/kg). (P) HE staining of hepatic lipid damage in Ash-*Thrb*-injected C57BL/6J mice treated with HFD or ART (20 mg/kg). (Q) Schematic of lipid metabolism mediated by thyroxine. Statistical comparisons were analyzed by one-way ANOVA analysis followed by Student's t-test. Data are presented as mean ± SD. ^**^p < 0.01.

We next searched for other underlying mechanisms of ART-mediated lipid-lowering effect. Since neuroendocrine system has been reported to play a crucial role in regulating energy metabolism, we then hypothesized that ART improved lipid metabolism via regulating hormones secretion 25. First, we detected the releases of lipid metabolism-associated hormones, including adrenaline, insulin, and glucagon 26. Here, ART did not significantly regulate the levels of these hormones (Figure S4A-S4C). In addition, our findings revealed a significant increase in the level of TRH in the PVN region of mice fed a HFD following treatment with ART (Figure 2A and S4D). Furthermore, our study also demonstrated that ART treatment resulted in a significant upregulation of serum levels of TSH, free total thyroxine (FT4) and free total triiodothyronine (FT3). These observations indicated that ART may improve HFD-induced lipid metabolism disorder through regulating neuroendocrine system (Figure 2B-2D and S4E-S4G). In particular, a proof-of-concept experiment using *l*-THY as a synthetic thyroid hormone was performed on LO2 cells. We found that PA or OA-induced lipid accumulation was significantly suppressed by *l*-THY (Figure S4H-S4I). Similarly, *l*-THY reduced aberrant lipid accumulation in PA or OA-induced HepG2 cells (Figure S4J-S4K), indicating an essential role of TH in controlling hepatic lipid metabolism.

To further confirm the association of ART-mediated TH release and lipid metabolism, we next used thiamazole (THI, a thyroid function inhibitor) to treat HFD-fed mice (Figure 2E). As shown in Figure 2F-2H, THI markedly reversed ART-regulated decrease of TC, TG, and non-HDL-C. Moreover, THI blocked ART-mediated inhibition in hepatic lipid accumulation (Figure 2I-2J and S5A-S5B). Meanwhile, we found THI obviously ablated the ART-decreased *Scd1*, and ART-increased *Cpt1a*, *Ppara* mRNA levels (Figure S5C).

Since thyroid hormone receptors (THRs) are important regulators of energy expenditure and hepatic lipid metabolism, we then tried to explore whether ART-induced TH release improved hepatic lipid metabolism via TH/THRs pathway. In this study, a liver-specific adeno-associated-virus-5-small-short-hairpin-RNA-TH receptor beta (Ash-*Thrb*) was developed to specifically target and reduce the expression of THRβ protein. As a result of *Thrb* knockdown, THRβ protein expression was successfully reduced (Figure 2K and S5D). Our data suggested that *Thrb* knockdown reversed ART-mediated down-regulation of TC, TG, and non-HDL-C (Figure 2L-2N) in Ash-*Thrb*-injected C57BL/6J mice. In addition, our findings indicate that ART treatment did not exhibit a significant inhibitory effect on hepatic lipid accumulation in the *Thrb* knockdown model (Figure 2O-2P and S5E-S5F). This observation suggests that the TH/THRs signaling pathway plays a crucial role in the regulation of hepatic lipid disorders by ART. Furthermore, we investigated the potential signaling mechanism of TH-mediated lipid metabolism using HepG2 cells. We found that *l*-THY significantly reduced PA-induced acetyl-histone H3 (K9) and acetyl-histone H4 (K16) in HepG2 cells by blocking the interaction between nuclear receptor corepressor 1 (NcoR1) and histone deacetylase 3 (HDAC3), which was previously reported as a fundamental biological pathway for lipid metabolism in livers (Figure S5G-S5H). Besides, *Thrb* knockdown suppressed *l*-THY-improved *Cpt1a* and *Ppara* mRNA levels (Figure S5I-S5K) in HepG2 cells, suggesting that TH regulated lipid metabolism through THR-dependent down-regulation of acetyl-histone. Taken together, our data indicate that ART may regulate hepatic lipid metabolism through TH/THR cascade (Figure 2Q).

### Hypothalamic Microglia-Mediated Neuroinflammation Contributes to TRH Neurons Injury for Neuroendocrine Control

The central regulation of thyroid hormones level by TRH neurons in hypothalamus has been widely recognized 27. Since microglial activation is highly associated with neuroinflammation and resultant neurons injury, we then speculated that ART may protect hypothalamic TRH neurons via inhibiting HFD-induced microglial activation 28.

**Figure 3:**
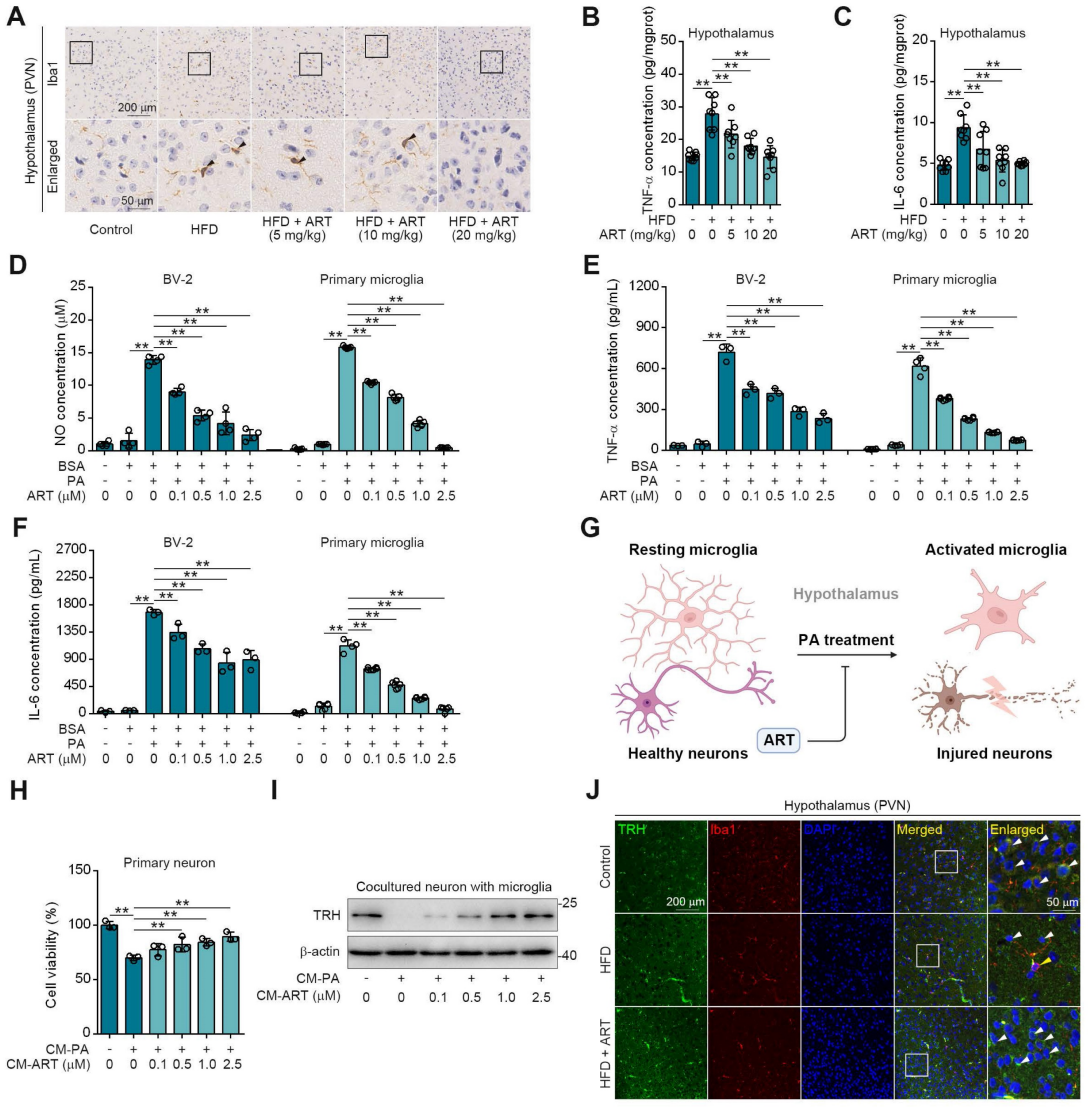
ART protects neurons from neuroinflammation in PVN region. (A) Iba1 immunohistochemical staining of PVN region in HFD-fed C57BL/6J mice treated with ART. (B-C) TNF-α (B), and IL-6 (C) assay of hypothalamus in HFD-fed C57BL/6J mice treated with ART (n = 8 mice each group). (D-F) NO (D), TNF-α (E), and IL-6 (F) assay of PA (100 μM)-induced BV-2 or primary microglia treated with ART (n = 3-4 each group). (G) Experimental design of neurons and microglia coculture. (H) Viability of CM-PA (100 μM)-induced primary neurons treated with CM-ART in hypothalamus (n = 3 each group). (I) Immunoblots of TRH from hypothalamus in CM-PA (100 μM)-induced primary neurons treated with CM-ART. (J) TRH and Iba1 immunofluorescence double staining of PVN region in HFD-fed C57BL/6J mice treated with ART. Statistical comparisons were analyzed by one-way ANOVA analysis followed by Student's t-test. Data are presented as mean ± SD. ^**^p < 0.01.

As expected, we observed a high Ionized calcium-binding adapter molecule 1 (Iba1) expression (a marker for microglia) in hypothalamic PVN of HFD-fed mice (Figure 3A), indicating that lipid metabolism disorder accelerated microglia-mediated neuroinflammation. However, ART significantly decreased Iba1 expression and improved microglial morphology (Figure 3A and S6A-S6G). Meanwhile, we also found that HFD-induced microglial inflammatory mediators (tumor necrosis factor-α, TNF-α and interleukin-6, IL-6) in hypothalamic PVN were decreased by ART (Figure 3B-3C and S6H-S6I). Immunofluorescence analysis in the PVN region revealed a colocalization of TNF-α/IL-6 with Iba1, suggesting that microglia predominantly contribute to the production of these inflammatory factors (Figure S6J-S6K). Furthermore, we found that ART significantly blocked nitrite oxide (NO), TNF-α, and IL-6 production in PA-induced BV-2 and primary microglia (Figure 3D-3F).

Given that microglia play a significant role in neuroinflammation-induced neuronal damage, we established an experimental model of primary neurons-microglia co-culture (Figure 3G and S6L). In Figure S6M-S6N, our findings revealed that the conditional medium (CM-PA) derived from microglia treated with PA significantly reduced the viability of neurons derived from PVN and suppressed the expression of TRH. Notably, this detrimental effect was not mitigated by direct treatment with ART. However, as shown in Figure 3H-3I, CM-PA treatment decreased neurons viability and TRH expression, which were significantly increased by PA/ART co-treated microglia-derived conditional medium (CM-ART). In vivo, we conducted immunofluorescence staining of TRH and Iba1 in PVN. We observed that the number of TRH-positive neurons around activated microglia (Iba1 positive) markedly decreased, which was improved effectively by ART treatment (Figure 3J and S6O-S6P). In addition, immunofluorescence staining of TRH and glial fibrillary acidic protein (GFAP) showed that ART did not exhibit an apparent effect on the co-expression of TRH and GFAP (a marker of astrocyte) (Figure S6Q). Collectively, these data demonstrate that microglia-mediated neuroinflammation plays a crucial role in ART-protected TRH neurons in hypothalamic PVN.

### PKCδ Serves as a Cellular Target against Microglial Activation

**Figure 4:**
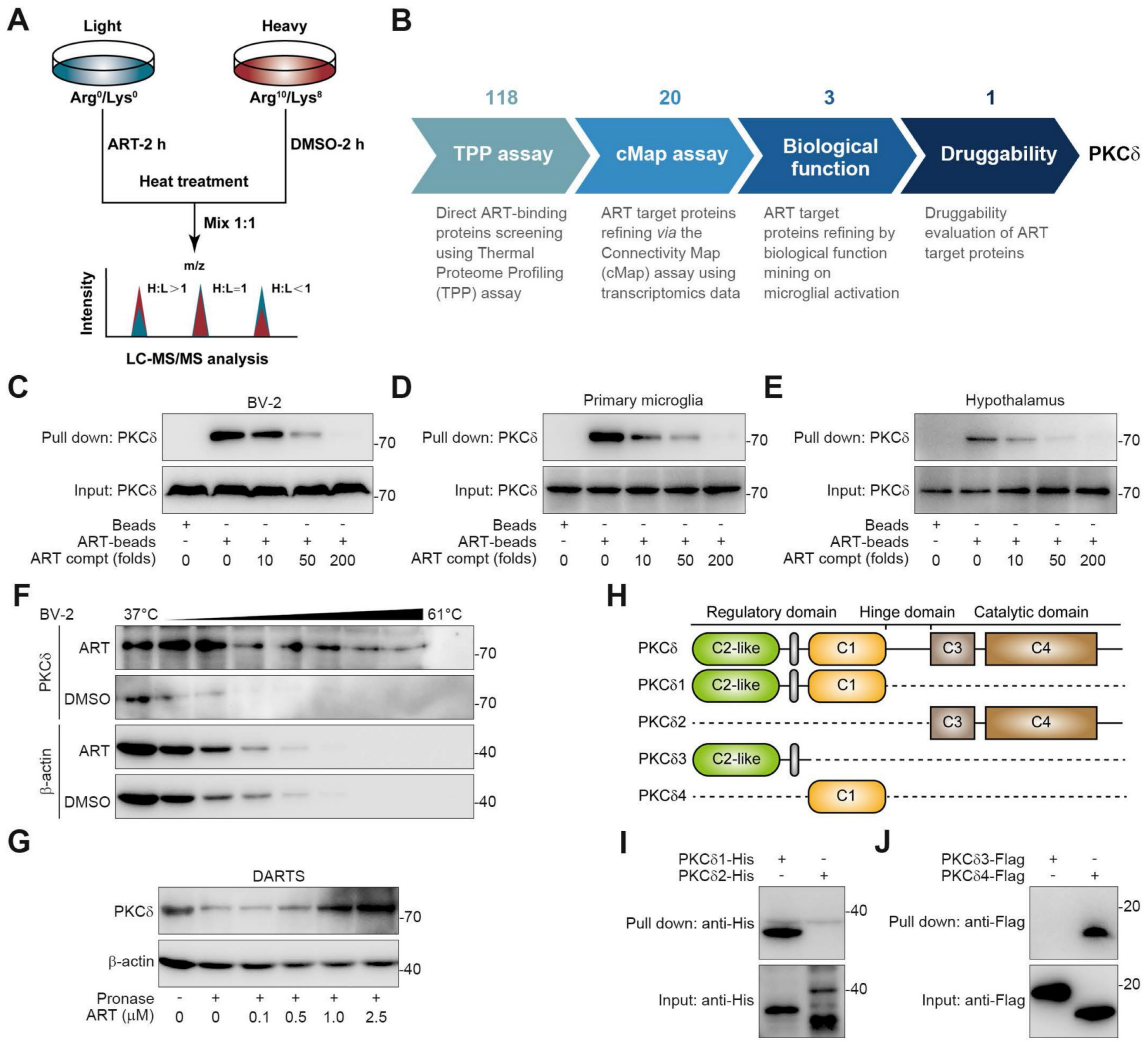
Discovery of PKCδ as the primary target of ART in BV-2 cells. (A) Workflow for target identification using TPP. (B) Flowsheet showing target evaluation of ART in TPP versus Connectivity Map. (C-E) BV-2 cell (C), primary microglia (D), or hypothalamus (E) lysates were incubated with ART-biotin followed by pull-down assay by streptavidin beads; immunoblots of PKCδ were shown. (F) BV-2 cells were treated with DMSO or ART (2.5 μM) and then heated at the indicated temperatures for 3 min; immunoblots of PKCδ were shown. (G) BV-2 cell lysates were incubated with ART and then lysates subjected to pronase digestion; immunoblots of PKCδ were shown. (H) Schematic diagram illustrating different truncations of PKCδ used. (I-J) 293T cells were transfected to express PKCδ1-His, PKCδ2-His, PKCδ3-HA or PKCδ4-HA; and cell lysates were incubated with ART-biotin followed by pull-down assay by using streptavidin beads; immunoblots of His or Flag were shown.

To explore the target proteins responsible for microglia-mediated neuroinflammation, we performed thermal proteome profiling (TPP) technology to identify the cellular targets of ART. Then, we totally identified 2292 potential ART-binding proteins by high performance liquid chromatography tandem mass spectrometry (HPLC-MS/MS), and 118 of them were confirmed according to experimental repeatability (Heavy/Light, H/L ratio) and *p* value (Figure 4A, Table S1). Out of the initial pool of candidates, a total of 20 potential target proteins were selected for further screening based on their significant contributions to the transcriptional expression profile (Figure S7A, Table S2). This selection process involved utilizing transcriptomics coupled with ConnectivityMap analysis. Next, biological function annotation revealed three major inflammation-associated proteins including protein kinase C δ (PKCδ), Serpinb1a and Serpinb6. In particular, PKC highlights a promising "druggability" in previous reports, hinting that ART may serve as a potential PKC inhibitor (Figure S7A). Next, NO assay with a pan-PKC inhibitor staurosporine (STAU) also verified PKC as a crucial target associated with neuroinflammatory pathology (Figure 4B and Figure S7B).

PKC family members are divided into multiple subtypes responsible for different biological functions 29. Besides PKCδ, it has been also reported that PKCα, PKCζ and PKCμ are highly associated with inflammation process 30-32. To explore the selective interaction of ART with PKCδ, we synthesized biotin-labelled ART probe (biotin-ART, Supplemental Information Reaction) for pull-down analysis. As shown in Figure 4C-4E and S7C, PKCδ was significantly pulled down by biotin-ART, which was blocked by excess amount of ART. However, ART did not show marked interaction with other PKCs, including PKCα, PKCζ, and PKCμ. In addition, we found that ART significantly prevented PKCδ protein degradation in cellular thermal shift assay (CETSA) experiment, but showed no effects on PKCα, PKCζ, and PKCμ (Figure 4F and S7D). Similarly, drug affinity responsive target stability (DARTS) analysis demonstrated that ART specially targeted PKCδ to inhibit its proteolysis against pronase (Figure 4G and S7E).

PKCδ consists of an N-terminal regulatory domain, a C-terminal catalytic domain, and a short ''hinge'' region 33. Regulatory domain contained C2-like region and C1 region, while catalytic domain contained C3 region and C4 region. In order to investigate which domain was specially targeted by ART, we truncated PKCδ into PKCδ1, PKCδ2, PKCδ3, and PKCδ4 (Figure 4H). Pull-down assay revealed that ART specifically interacted with the distinctive C1 domain, but not the C2-like, C3 or C4 domains (Figure 4I-4J). In sum, our data suggest that PKCδ serves as an essential cellular target of ART in microglia.

### PKCδ Senses Lipid by Palmitoylation Modification for Microglial Activation

**Figure 5:**
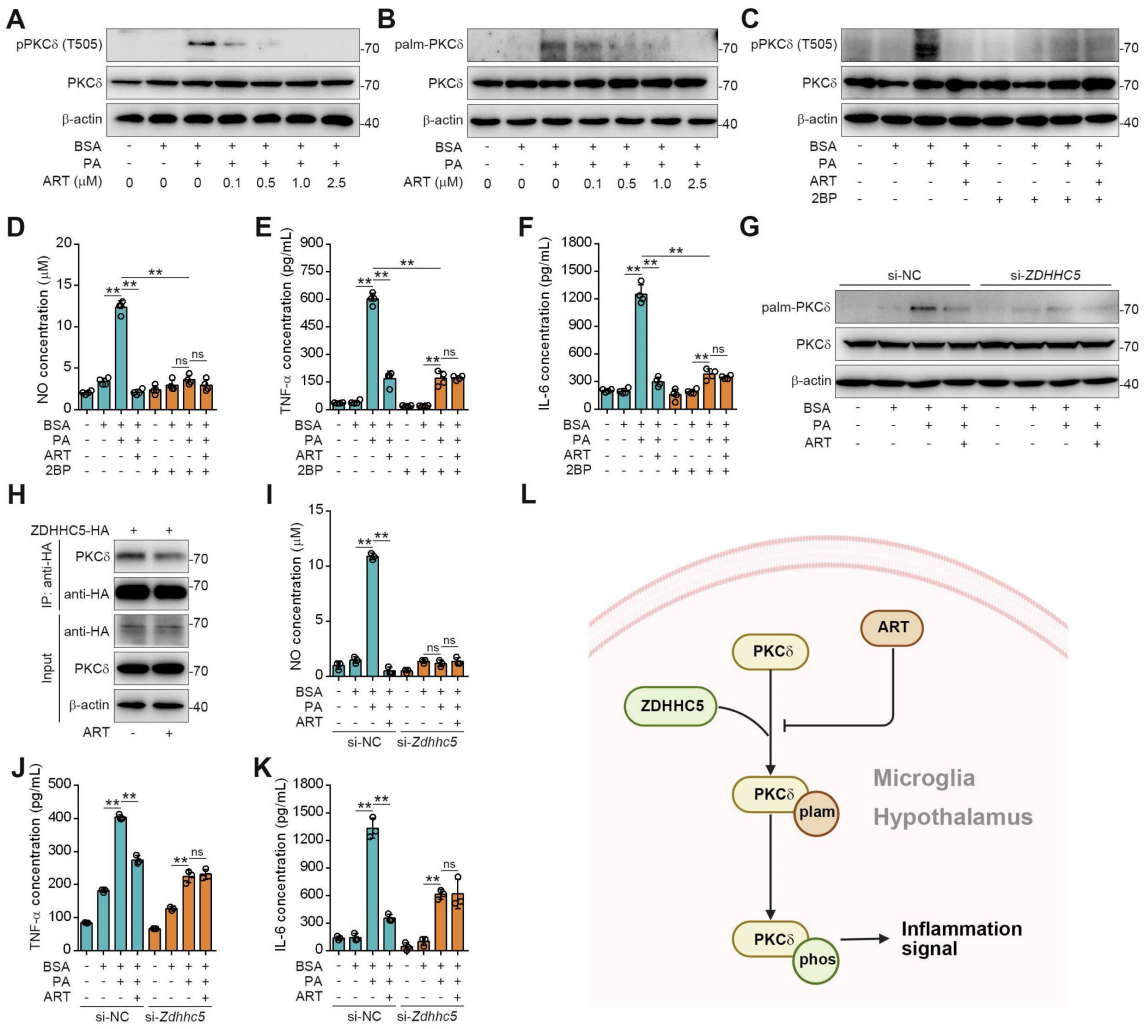
ART inhibits PKCδ phosphorylation by blocking PKCδ palmitoylation to attenuate neuroinflammation. (A) Immunoblots of pPKCδ and PKCδ in PA (100 μM)-induced BV-2 cells treated with ART (2.5 μM). (B) Immunoblots of palm-PKCδ and PKCδ in PA (100 μM)-induced BV-2 cells treated with ART (2.5 μM). (C) Immunoblots of pPKCδ and PKCδ in PA (100 μM)-induced BV-2 cells treated with ART (2.5 μM) or 2BP (1 μM). (D-F) NO (D), TNF-α (E), and IL-6 (F) assay of PA (100 μM)-induced BV-2 cells treated with ART (2.5 μM) or 2BP (1 μM) (n = 4 each group). (G) BV-2 cells were transfected to express si-NC or si-*Zdhhc5* and treated with PA (100 μM) or ART (2.5 μM); immunoblots of palm-PKCδ and PKCδ were shown. (H) 293T cells were transfected to express ZDHHC5-HA and treated with 2.5 μM ART; immunoblots of HA, PKCδ and β-actin using anti-HA agarose beads were shown. (I-K) BV-2 cells were transfected to express si-NC or si-*Zdhhc5* and treated with PA (100 μM) or ART (2.5 μM); NO (I), TNF-α (J), and IL-6 (K) assay were shown (n = 3 each group). (L) Schematic of ZDHHC5, PKCδ phosphorylation and palmitoylation. Statistical comparisons were analyzed by one-way ANOVA analysis followed by Student's t-test. Data are presented as mean ± SD. ^**^p < 0.01.

Since PKCδ activation serves as a key prerequisite for neuroinflammation, we then explored the regulation mechanism of ART on PKCδ function 34. Recent study has shown that protein palmitoylation played an essential role for phosphorylation regulation, and the covalent attachment of PA to cysteine residues (S-palmitoylation) is a widespread modification on proteins 35-36. First, we observed that PA treatment significantly increased PKCδ phosphorylation in microglia, which was reversed by ART (Figure 5A). Here, we speculated that PKCδ phosphorylation may be potentially regulated via its prior palmitoylation. To this end, we conducted acyl-biotinyl exchange analysis and observed that PA significantly increased PKCδ palmitoylation in microglia, which was reversed by ART (Figure 5B and S8A). Additionally, an evident increase in PKCδ palmitoylation was observed in the hypothalamus of mice fed a HFD. In particular, we found that PKCδ palmitoylation in hypothalamus was markedly blocked by ART treatment (Figure S8B-S8C). Next, we used 2BP, a protein palmitoylation blocker, to inhibit PKCδ palmitoylation in PA-induced BV-2 cells (Figure S8D). As shown in Figure 5C, 2BP almost completely abolished ART-mediated PKCδ phosphorylation inhibition. Furthermore, we observed that 2BP treatment significantly reversed ART-mediated inhibition of NO, TNF-α, and IL-6 releases (Figure 5D-5F), indicating that PKCδ palmitoylation plays a crucial role in lipid-induced microglial activation and neuroinflammation.

To investigate the molecular mechanism of PKCδ palmitoylation, we performed global proteomics analysis and identified ZDHHC5 as the most significant palmitoyltransferase in hypothalamus by HPLC-MS/MS (Table S3). We then knocked down ZDHHC5 and found that PA-induced PKCδ palmitoylation was significantly weakened in *Zdhhc5* knockdown cells. Meanwhile, ART-mediated suppression of PKCδ phosphorylation was also remarkably blocked by *Zdhhc5* knockdown (Figure 5G, Figure S8E), indicating that ZDHHC5-dependent PKCδ palmitoylation contributes to PKCδ phosphorylation. Co-IP experiment also revealed that PKCδ-ZDHHC5 interaction could be pharmacologically inhibited by ART (Figure 5H). Furthermore, ART lost its inhibitory effect on NO, TNF-α, and IL-6 release upon si-*Zdhhc5* treatment (Figure 5I-5K). Thus, these results reveal that ZDHHC5-dependent palmitoylation is highly involved in PKCδ activation in microglia (Figure 5L).

We next investigated PKCδ-dependent neuroinflammation signaling pathways. As shown in Figure S9A-S9B, 3 major inflammatory signaling pathways were enriched by KEGG pathway analysis including NLRP3, NF-κB, and Janus kinase-signal transduce-activator of transcription (Jak-Stat). Next, western blotting was performed to investigate the effect of ART on NLRP3 inflammasome pathway. As shown in Figure S9C, PA increased NLRP3, caspase1, IL-1β and IL-18 levels, which were markedly reversed by ART. Moreover, a marked increase of inhibitor of κB kinase β phosphorylation (pIKKβ), pNF-κB p65 and inhibitor of nuclear factor κBα phosphorylation (pIκBα) expressions was detected in PA group, which was suppressed by ART (Figure S9D). Furthermore, we observed that PA induced an increase in phosphorylation levels of Jak2, Stat5 and Stat3, which was blocked by ART treatment (Figure S9E). Taken together, our data suggest that PKCδ serves as a lipid sensor for microglial activation and neuroinflammation via unique palmitoylation modification.

### Pharmacologically Targeting Microglial PKCδ in Hypothalamus Alleviates Hepatic Lipid Metabolism Disorder *in vivo*

First, we investigated whether pharmacologically targeting PKCδ in hypothalamus exhibited an anti-neuroinflammation effect. The *Prkcd*^+/-^ mouse model was established by CRISPR/Cas9 (Figure S10A). We found that ART exerted an obvious suppression on Iba1 expression in PVN of HFD-fed *Prkcd*^+/+^ mice, which was significantly reversed in *Prkcd*^+/-^ mice (Figure S10B-S10E). Moreover, ART blocked TNF-α and IL-6 release in PVN of HFD-fed *Prkcd*^+/+^ mice, but showed less effect in *Prkcd*^+/-^ mice (Figure S10F-S10G). Furthermore, pharmacokinetic analysis of the blood-brain barrier (BBB) transport of ART revealed that ART could pass through BBB and accumulated in hypothalamus (S10H). These data indicate that ART may exert an anti-neuroinflammation effect in hypothalamus through targeting PKCδ.

**Figure 6:**
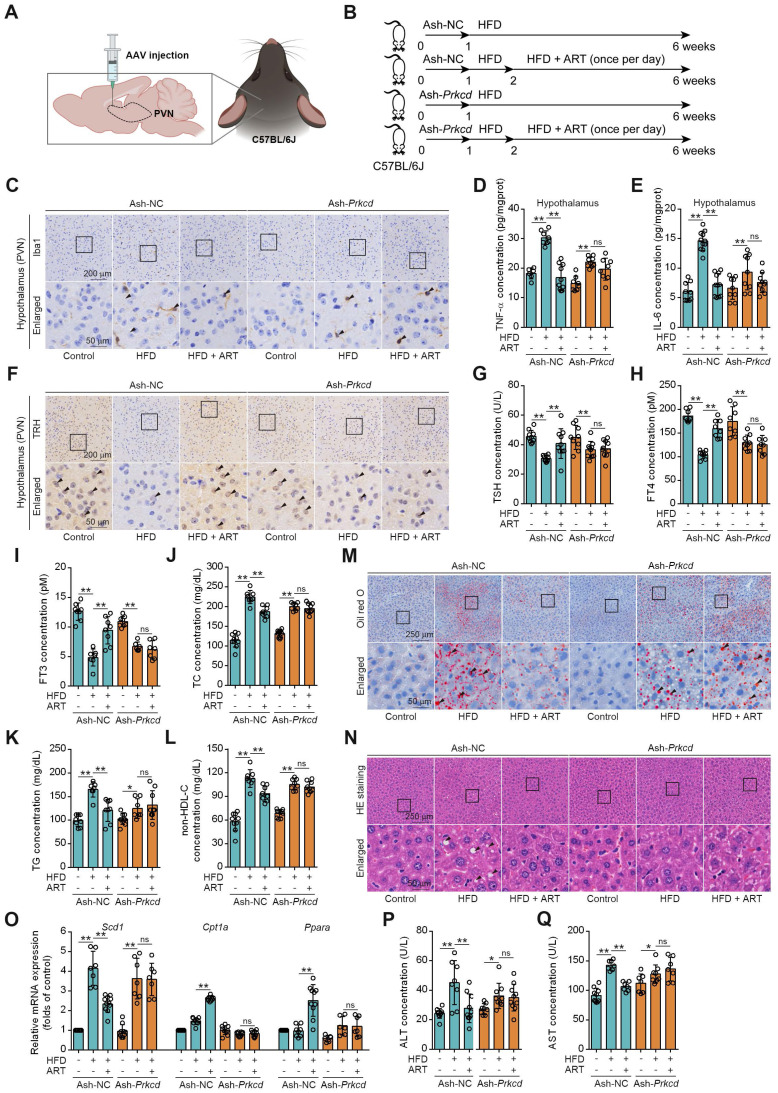
*Prkcd* knockdown of PVN region reverses ART suppression on HFD-fed hepatic lipid metabolism disorder in C57BL/6J mice. (A) Schematic depicting intracerebroventricular AAV injections. (B) Experimental design of ART treatment in Ash-*Prkcd*-injected C57BL/6J mice fed on HFD. (C) Iba1 immunohistochemical staining of PVN region in Ash-*Prkcd*-injected C57BL/6J mice treated with HFD or ART (20 mg/kg). (D-E) TNF-α (D), and IL-6 (E) assay of hypothalamus in Ash-*Prkcd*-injected C57BL/6J mice treated with HFD or ART (20 mg/kg) (n = 6-10 mice each group). (F) TRH immunohistochemical staining of PVN region in Ash-*Prkcd*-injected C57BL/6J mice treated with HFD or ART (20 mg/kg). (G-I) TSH (G), FT4 (H), and FT3 (I) assay of Ash-*Prkcd*-injected C57BL/6J mice treated with HFD or ART (20 mg/kg) in serum (n = 7-9 mice each group). (J-L) TC (J), TG (K), and non-HDL-C (L) assay of Ash-*Prkcd*-injected C57BL/6J mice treated with HFD or ART (20 mg/kg) in serum (n = 7-10 mice each group). (M) Oil red O staining of hepatic lipid accumulation in Ash-*Prkcd*-injected C57BL/6J mice treated with HFD or ART (20 mg/kg). (N) HE staining of hepatic lipid damage in Ash-*Prkcd*-injected C57BL/6J mice treated with HFD or ART (20 mg/kg). (O) *Scd1*, *Cpt1a* and *Ppara* mRNA levels of Ash-*Prkcd*-injected C57BL/6J mice treated with HFD or ART (20 mg/kg) (n = 6-10 mice each group). (P-Q) ALT (P), and AST (Q) assay of Ash-*Prkcd*-injected C57BL/6J mice treated with HFD or ART in serum (20 mg/kg) (n = 7-10 mice each group). Statistical comparisons were analyzed by one-way ANOVA analysis followed by Student's t-test. Data are presented as mean ± SD.^ *^p < 0.05, ^**^p < 0.01.

To further confirm whether ART inhibited hypothalamic neuroinflammation by selectively targeting PKCδ in microglia of PVN, a microglia-specific adeno-associated-virus-5-small-short-hairpin-RNA-*Prkcd* (Ash-*Prkcd*) was designed for stereotaxic intracranial injection into PVN of mice (Figure 6A-6B). The administration of Ash-*Prkcd* injection effectively decreased the expression of PKCδ in microglia located in the PVN region (Figure S10I). Similarly, we also found that ART effectively reduced HFD-induced Iba1 production in Ash-negative-control (Ash-NC)-injected group, which was not obviously affected by ART in Ash-*Prkcd*-injected group (Figure 6C and S10J-S10L). In addition, ART markedly reduced hypothalamic inflammatory mediator in Ash-NC-injected group including TNF-α and IL-6, which was effectively reversed in Ash-*Prkcd*-injected mice (Figure 6D-6E). Taken together, our data suggest that ART selectively targets microglial PKCδ in hypothalamus *in vivo* to suppress neuroinflammation.

Then, we attempted to test whether pharmacologically targeting PKCδ exerted lipid-lowering effect. As shown in Figure 6F-6I and S10M, ART markedly increased TRH neurons number in NC-injected PVN, thereby promoting thyroid hormone production. However, similar effect was not observed in Ash-*Prkcd*-injected mice. Moreover, HFD-induced TC, TG, and non-HDL-C levels were significantly ameliorated by ART in Ash-NC-injected mice, while ART treatment did not display similar therapeutics in Ash-*Prkcd*-injected mice (Figure 6J-6L). Meanwhile, ART improved HFD-induced hepatic steatosis and lipid metabolism-related gene expressions in Ash-NC-injected mice; however, Ash-*Prkcd* injection reversed ART-dependent therapeutic effects (Figure 6M-6O and S10N-S10O). Furthermore, ART decreased ALT and AST levels in Ash-NC-injected mice, which was also ablated in Ash-*Prkcd*-injected mice (Figure 6P-6Q). To exclude the direct effect of PKCδ on lipid metabolism of hepatocytes, we then down-regulated PKCδ expression in HepG2 and LO2 cells, and found that ART did not show obvious regulation effect on lipid accumulation (Figure S11A-S11D). Collectively, our results suggest that microglial PKCδ in hypothalamus represents a crucial target for alleviating hepatic lipid disorder *in vivo*.

### PKCδ is Correlated with the Microglial Activation in the Brains of Hyperlipemia Patients

**Figure 7:**
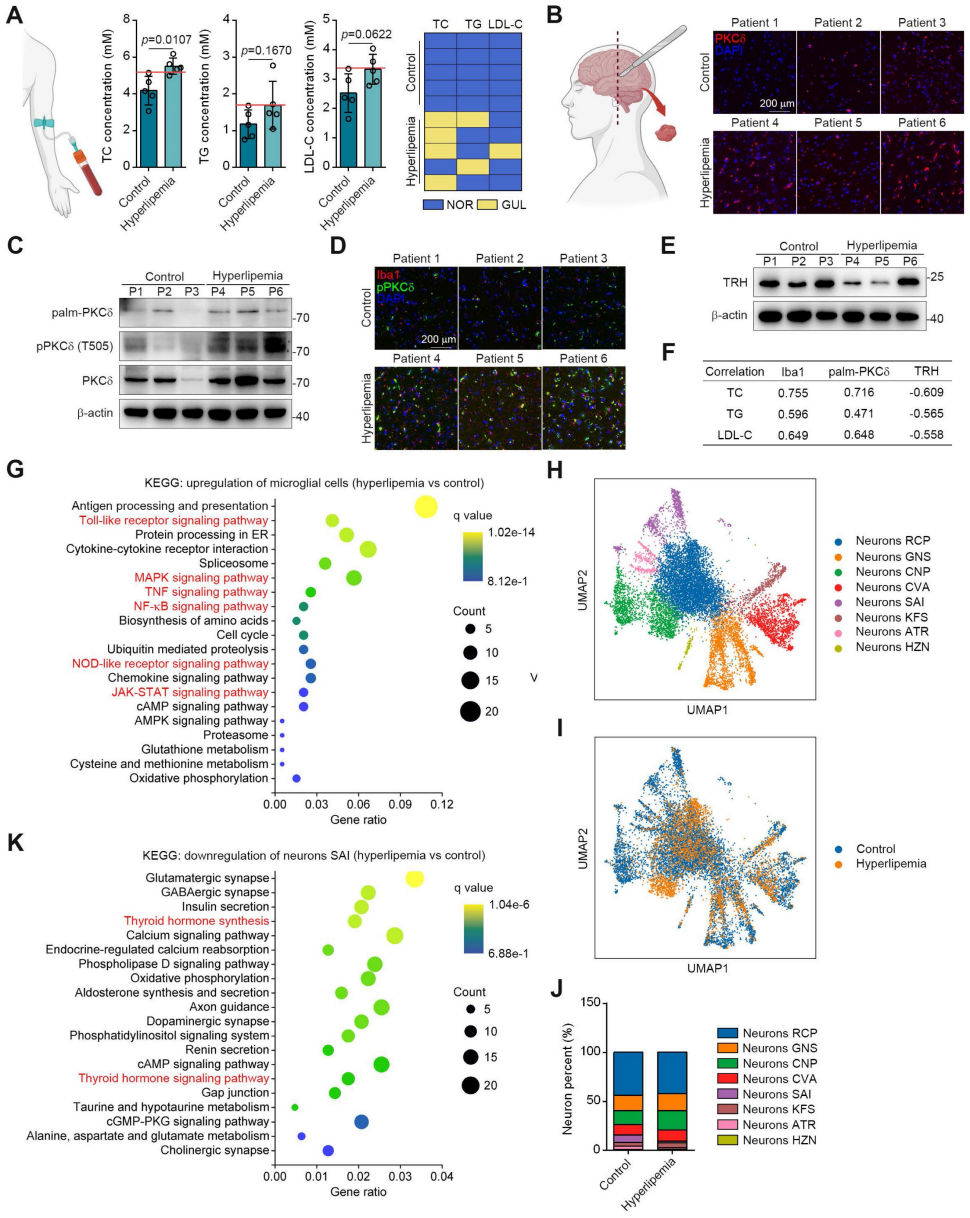
PKCδ expression is upregulated in fatty brain and correlates with hyperlipemia progression. (A) TC, TG and LDL-C assay of indicated individuals, blue panels mean blood lipids are normal (NOR); yellow panels mean blood lipids are greater than the upper limit of normal values (GUL, n = 5 individuals each group). (B) Immunofluorescence staining for PKCδ on brain sections of indicated individuals. (C) Immunoblots of palm-PKCδ, pPKCδ and PKCδ in brain sections of indicated individuals. (D) pPKCδ and Iba1 immunofluorescence double staining on brain sections of indicated individuals. (E) Immunoblots of TRH in brain sections of indicated individuals. (F) Pearson correlation analysis of serum lipid (TC, TG and LDL) and Iba1, palm-PKCδ, TRH expressions (Western blotting). (G) KEGG analysis of top 20 up-regulated pathways of microglial cells hyperlipemia vs control). (H) UMAP plot of single-nuclei transcriptomic data from neurons of all patient samples. (I) Comparison of UMAP plot between control and hyperlipemia neurons. (J) Cell percentage of single-nuclei transcriptomic data in neurons. (K) KEGG analysis of top 20 down-regulated pathways of neurons SAI (hyperlipemia vs control).

To translate our findings, we obtained the brain tissues and serum samples from 5 control donors and 5 individuals with hyperlipemia (Figure 7A). Immunohistochemistry and western blotting results showed that PKCδ, pPKCδ and plam-PKCδ levels were all higher in the brains of hyperlipemia patients (Figure 7B-7C). Furthermore, immunofluorescent analysis demonstrated a significant increase in the colocalization of pPKCδ with Iba1 in brains with hyperlipemia. This finding suggests a positive correlation between PKCδ activation and microglial activation (Figure 3D). Meanwhile, we confirmed the decreased TRH levels in hyperlipemia brains (Figure 7E). Importantly, we also observed a trend but no statistical significance, due to high interindividual variability and a limited number of subjects that TC, TG and LDL-C levels in hyperlipemia patients positively correlated with Iba1 and plam-PKCδ expressions (Figure 7F). Furthermore, TRH level negatively correlated with hyperlipemia severity (Figure 7F). Together, these data indicate that PKCδ-mediated neuroinflammation may be linked to lipid metabolism disorder.

Next, we conducted single-nuclei RNA-seq to characterize cell types in brain tissue (Figure S12A-S12B). As shown in Figure S12C-S12D, microglial and neuronal cell clusters presented marked changes in UMAP plots and cell percent between control and patient. These data showed consistently increased PKCδ-associated inflammatory signatures in the microglia of hyperlipemia brains, such as Toll-like receptor, NF-κB, mitogen activated kinase-like protein (MAPK), NOD-like receptor and JAK-STAT pathways (Figure 7G). In neurons, Kyoto encyclopedia of genes and genomes (KEGG) pathway analysis suggested several common neuron survival pathways were down-regulated, which were highly associated with glutamatergic, cholinergic, GABAergic, and dopaminergic synapse function. Besides, some neuronal apoptosis pathways such as reactive oxygen species signaling cascade were also up-regulated (Figure S12E-S12F).

To further conduct detailed transcriptomic analysis of TRH neurons, we carried out the unbiased clustering in the neuronal population. Our result revealed 8 neuron clusters (Figure 7H and S12G). Among the clusters, neurons SAI highly expressed *TRH* genes and the cell percent decreased markedly in hyperlipemia brain (Figure 7I-7J and S12H). Further KEGG analysis of neurons SAI revealed that TH synthesis was decreased and TH signaling pathway was down-regulated, suggesting that PKCδ-mediated microglial activation may specially cause TRH neurons damage in hyperlipemia brain (Figure 7K). Collectively, these data suggest a clinical association of microglial PKCδ activation in brain with peripheral lipid metabolism disorder.

## Discussion

Currently, statins attenuate lipid metabolism disorder mainly through targeting HMG-CoA reductase in hepatocytes 37. Moreover, fibrates induce lipoprotein lipase expression and promote lipid metabolism by activating PPAR 38. Interestingly, our study has provided intriguing evidence suggesting that ART may regulate hepatic lipid metabolism via the hypothalamus-liver axis, which represents a completely different mechanism. Furthermore, the FDA has granted approval to ART, which has subsequently gained widespread utilization in clinical practice. Even at high doses, ART has demonstrated a commendable safety profile, as it did not result in significant adverse effects such as elevated transaminase levels, gastrointestinal symptoms, rhabdomyolysis, or rash 39. The short terminal elimination half-life of ART suggests that prolonged administration of the drug does not result in significant drug accumulation 40. Consequently, our findings present a novel avenue for the development of therapeutics targeting metabolic diseases. Meanwhile, the results of our study indicate that there was no significant impact of ART on diet, weight loss, or other symptoms associated with lipid metabolism disorders, indicating a clinically acceptable therapeutic regimen to treat metabolic syndrome in the future 41.

A growing body of literature supports the notion that neuroinflammation plays a pivotal role in the pathogenesis of numerous neurological disorders, including Alzheimer's disease (AD), Parkinson's disease (PD), and amyotrophic lateral sclerosis (ALS) 42-43. This study revealed a significant association between hypothalamic inflammation and hepatic lipid metabolism, indicating the regulatory role of neuroendocrine signals. Therefore, pharmacological inhibition of hypothalamic neuroinflammation is of great significance to the treatment of lipid metabolism disorder. Besides adrenaline, insulin, and glucagon also function as master regulators of lipid metabolism. However, in our study, the administration of ART did not result in any noticeable impact on the hormone levels, suggesting that the regulation of peripheral lipid metabolism by the hypothalamic TRH neurons-dependent TH signal may operate independently. Particularly, previous reports show that several anti-neuroinflammation drugs exhibit an efficient antihyperlipidemic effect such as baicalein, resveratrol and berberine, which may stimulate our interests to further explore whether these agents are potentially involved in the neuroinflammation progress for improving lipid metabolism disorder 44-46.

Palmitoylation is one of major protein post-translational modifications and highly involved in multiple pathological processes 47-49. Previous studies indicated that protein palmitoylation was a prerequisite for phosphorylation 35.

Our data suggested that PKCδ phosphorylation was markedly regulated by PKCδ palmitoylation, thereby causing subsequent microglial activation pathways in hypothalamus. These observations indicate that palmitoylation contributes to microglial activation and may function as a potential pharmacological target. Structurally, PKCδ is composed of C1 and C2 domains 50. Given that ART selectively interacted with the C1 domain of PKCδ, we then speculated that ART may reduce PKCδ palmitoylation via blocking palmitoyltransferase ZDHHC5 binding to C1 region, subsequently inhibiting PKCδ phosphorylation in catalytic domain. Therefore, the palmitoylation of PKCδ may serve as a pivotal biological event in microglial activation, which in turn influences peripheral lipid metabolism.

Due to ethical limitations, we were only able to obtain samples of the patient's cortical tissue for analysis in our study. Nevertheless, our observations consistently revealed a notable correlation between heightened serum lipid levels and the activation of microglial cells, accompanied by a decline in TRH levels within the brain. These findings align with previous studies, substantiating the notion that hyperlipidemia is a contributing factor to augmented PKCδ expression in brain tissue 51-53. Of note, this observation was further supported by our analysis of single-cell transcriptomics, with a specific focus on the SAI neuronal cluster that highly expressed *TRH* genes. These data provide a crucial clue of clinical translational value by targeting neuroendocrine system to regulate lipid metabolism disorder in humans. Further, it suggests the possibility of repurposing previous Food and Drug Administration (FDA)-approved drugs in central nervous system for intervention in lipid metabolism disorders.

## Conclusion

In summary, we have provided proof-of-concept evidence that microglial PKCδ in the hypothalamus regulates lipid metabolism disorder through neuroendocrine signals. Particularly, these findings indicate that targeting microglial PKCδ could be a promising therapeutic approach for treating fatty liver disease, which is distinct from current treatment strategies.

## Experimental Section/Methods

### Chemicals and reagents

Artemether (C_16_H_26_O_5_, molecular mass 298.37), Dihydroartemisinin (C_15_H_24_O_5_, molecular mass 284.35) and Artesunate (C_19_H_28_O_8_, molecular mass 384.42) were purchased from KPC Pharmaceuticals (Kunming, Yunnan, China). Thiamazole (#BD220076), *l*-thyroxine sodium (#BD105752), N-ethylmaleimide (NEM, #BD144276), nile red (#BD306389), and cholesterol (CHO, BD110544) were purchased from Bidepharm (Shanghai, China). 2-bromopalmitate (2BP, #R029267) was purchased from Rhawn (Shanghai, China). Hydroxylamine (HA, #ALD-H164487) solution was purchased from Aladdin (Shanghai, China). 3-(4, 5-dimethyl thiazol-2-yl)-2, 5-diphenyl tetrazolium bromide (MTT, #88417) was purchased from Sigma (St. Louis, MO, USA). EZ-link® N-[6-(biotinamido) hexyl]-3′-(2′-pyridyldithio)propionamide (biotin-HPDP, #A8008) was purchased from Apexbio (Houston, TX, USA). Staurosporine (#HY-15141) was purchased from MedChem Express (Shanghai, China). Biotin-PEG_3_-CH_2_CH_2_COOH (#P004009) was purchased from ToYongBio (Shanghai, China). Palmitic acid (PA, #P0002), stearic acid (SA, #S0163), and oleic acid (OA, #O0180) were purchased from Tokyo Chemical Industry (Tokyo, Japan). Bovine serum albumin (BSA, #BAH66) was purchased from Equitech-Bio (Shanghai, China). Polyvinylidene difluoride (PVDF, #IPVH00010) membrane was purchased from Merck Millipore (Bedford, MA, USA). Antibodies against Stat3 (#12640), Stat5 (#94205), Jak2 (#3230), p-Stat3 (#4113), p-Stat5 (#4322), p-Jak2 (#4406), IKKβ (#8943), IκBα (#4812), NF-κB p65 (#8242), p-IKKβ (#2697), p-IκBα (9246), p-NF-κB p65 (#3033), procaspase1 (#2225), caspase1 (#67314), proIL-1β (12242), IL-1β (#52718), NLRP3 (#15101), PKCα (#2056), PKCδ (#9616), PKCζ (#9368), PKCμ (#90039), p-PKCδ (#9374), Histone H3 (#4499), Histone H3 (acetyl K9, #9649), Histone H4 (#13919), HA tag (#11846), SCD1 (#2794), β-actin (#3700), rabbit IgG (#7074) and mouse IgG (#7076) were purchased from Cell Signaling Technology (Danvers, MA, USA). Antibodies against IL-18 (#ab71495), Iba1 (#ab283319), TRH (#ab171958), PKCδ (#ab182126), p-PKCδ (#ab60992), THRβ (#ab155297), Histone H4 (acetyl K16, #ab109463) and GFAP (#ab279290) were obtained from Abcam (Boston, MA, USA). Antibodies against HDAC3 (#10255-1-AP), NcoRI (#20018-1-AP), PPARA (#66826-1-Ig), CPT1A (#15184-1-AP), THRβ (#21007-1-AP), TNF-α (#60291-1-Ig), IL-6 (#21865-1-AP) and Iba1 (#10904-1-AP) were obtained from Proteintech (Rosemont, IL, USA).

### Human study and approval

The study was approved by the Research Ethics Committee of Peking University Third Hospital (Approval No. M2022433). A total of 10 adults who underwent serum biochemical analysis for suspected dyslipidemia were enrolled for analysis. Normal brain specimens were obtained during brain excision surgery due to glioma. Written informed consent was obtained from each participant for collection of samples.

### Mice study and approval

C57BL/6J and Balb/c mice were procured from VITAL RIVER Laboratories located in Beijing, China. Throughout the experimental period, these mice were provided *ad libitum* access to both food and water. They were housed under specific pathogen-free conditions, maintaining a controlled environment with a 12 h cycle of light and darkness, with a constant temperature of 25 °C. All experimental procedures undertaken adhered strictly to the Animal Welfare Standard guidelines and were granted approval by the Biomedical Ethics Committee of Peking University (Approval No. LA2020023).

### Generation of *Prkcd*^+/-^ mice

Exons 2-14 of the mouse *Prkcd* gene were targeted because of sequence specificity against other PKC (Cyagen, Shanghai, China). Homologous recombination-based technique was performed to construct a targeting vector by a bacterial artificial chromosome clone. Short homology arm extended 1.2 kb to 5′ of loxP/flippase recognition target (FRT)-flanked Neo cassette, while long homology arm began on the 5′ single loxP side (about 6.5 kb long). Then, loxP/FRT-flanked Neo cassette was used to change the exons 2-14 of *Prkcd*. Embryonic stem cells (derived from iTL BA1 hybrid [C57BL/6J] strain) after undergone homologous recombination were acquired and wild-type C57BL/6J mice were crossed with chimeric mice to obtain F1. Ayu1-Cre mice in C57BL/6J background were mated with F1 mice to generate *Prkcd*^+/-^ mice (homozygous death).

### Viral constructs and injections

*Prkcd*-specific small short hairpin RNA (shRNA) was cloned and packaged into an adeno-associated virus 5 (AAV5) with the microglia-specific CD68 promoter (Hanbio, Shanghai, China). *Thrb*-specific shRNA was cloned and packaged into an adeno-associated virus 5 (AAV5) with the hepatocyte-specific TBG promoter (Hanbio, Shanghai, China). The AAV5-shRNA-NC/*Prkcd* (Ash-NC/*Prkcd*) or Ash-NC/*Thrb* was packaged with pAAV-RC and pHelper using the triple-plasmid transient transfection method (HB infusionTM Kit; Hanbio, Shanghai, China). Viral particles were applied in the following animal experiments after purification. The shRNA-*Prkcd* sequence was as follows: 5′-GGGAUUAAAGUGUGAAGAUTT-3′. The shRNA-*Thrb* sequence was as follows: 5′-GGAGGAGAAGAAAUGUAAATT-3′.

For Ash-NC/*Prkcd* injection into the PVN, eight-week-old male C57BL/6J mice were anesthetized with isoflurane inhalation (Harvard Apparatus, Holliston, MA, USA) and placed in a stereotaxic frame (RWD, Shenzhen, Guangdong, China). 100 nL virus were administered using a 33-gauge needle connected to a 5 μL syringe at 20 nL/min according to the following coordinates: -0.8 mm below the surface of the skull, ± 0.25 mm lateral to midline, and -4.6 mm posterior to the Bregma. Before needle retraction, a 10-min time lapse was allowed. After the procedure, the incision was sutured and mice were placed in a heated cage until they recovered from anesthesia. Experiments were conducted at least 2 weeks after injections to ensure AAV expression.

For Ash-NC/*Thrb* injection, eight-week-old male C57BL/6J mice were anesthetized with isoflurane inhalation (Harvard Apparatus, Holliston, MA, USA) and 100 nL virus in 100 μL of 0.9% NaCl were injected into mice* via* the tail vein. Experiments were conducted at least 2 weeks after injections to ensure AAV expression.

### HFD feeding and pharmacological treatment

Mice were randomly divided into control group, HFD group, and pharmacological treatment group. HFD group and pharmacological treatment group were fed with HFD (10% lard, 10% vitelline, 1% cholesterol, 0.2% sodium cholate and 78.8% common diet) for 5 weeks to induce LMD while control group was cultured with common diet. From the 8th day of HFD treatment, oral administration with ART (5, 10 or 20 mg/kg body weight), THI (a thyroid hormone inhibitor, 15 mg/kg body weight) or *l*-THY (a positive drug for treating hyperlipidemia, 1 mg/kg body weight) was performed once per day. After treatment for 4 weeks, the mice were sacrificed for analysis.

### Cell lines

BV-2, LO2, HepG2, or Caco-2 cells (Cell Bank of Peking Union Medical College, Beijing, China) were cultured in Dulbecco's modified eagle medium (DMEM, #CM15019, MACGENE, Beijing, China) containing 10% fetal bovine serum (FBS, #AB-FBS0500S, BioRuler, Beijing, China), 100 U/mL penicillin, and 100 μg/mL streptomycin (#CC004, MACGENE, Beijing, China). Cell culture condition was kept at 37 °C under 95% absolute humidity in a 5% CO_2_ incubator.

Primary microglia were dissociated from neonatal mouse brains as previously described 54. Briefly, hypothalamus was mechanically isolated and meninges was stripped with a 70 μm filter. Cell suspensions were prepared with trypsin and pooled in DMEM-F12 (#CM10090, MACGENE, Beijing, China) supplemented with 10% fetal bovine serum, 100 U/mL penicillin, and 100 μg/mL streptomycin. Cellular debris was removed by changing culture media the next day. After 2-3 weeks of culture, primary microglia were collected by shaking the flask at 100 rpm for 30 min.

Primary cultures of hypothalamic neurons were derived from the hypothalamus of 18-day-old fetal mice. All plates and chamber slides were coated with 100 μg/mL poly-D-lysine (#P8140, Solarbio, Beijing, China), and washed with sterile phosphate buffered saline (PBS) before plating. Isolated hypothalamus were collected in one 50 mL tube and 0.05% trypsin was added to the tissue. The tissue/trypsin mixture was incubated in a 37 °C incubator for 15 min with inverting 3 times every 5 min and DMEM containing 10% FBS was used to stop digestion. The supernatant was centrifuged at 1000 rpm for 2 min and cell pellet was collected and re-suspended in DMEM containing 10% FBS. Cells were diluted and plated for 6 h, and the culture medium was changed by serum free SFM neurobasal medium (#21103049, Invitrogen, Carlsbad, CA, USA) containing B-27 supplement (#17504044, Invitrogen, Carlsbad, CA, USA). Primary hypothalamic neurons were cultured for 14 d before experiments.

Primary hepatocytes were obtained from 10-week-old male C57BL/6J mice. The liver was perfused via the inferior vena cava with 30 mL of Hank's Balanced Salt Solution (HBSS) at a flow rate of 5 mL/min. The perfusate was allowed to outflow via the hepatic portal vein. This was followed by perfusion with 50 mL of HBSS containing 0.05% collagenase type IV (#17104019, Invitrogen, Carlsbad, CA, USA) and 5 mM CaCl_2_. All operations were conducted at a temperature of 37 °C. The livers were subsequently collected and transferred into RPMI 1640 medium (#PM150110, Procell, Wuhan, Hubei, China) containing 2% fetal bovine serum. The livers were gently stirred at a temperature of 4 °C to facilitate the separation of hepatocytes. After passing through a 100 μm strainer, the hepatocytes were washed twice with PBS and subsequently cultured in RPMI 1640 medium supplemented with 10% fetal bovine serum, 100 U/mL penicillin, and 100 μg/mL streptomycin 55.

### Cell treatment

BV-2 cells or primary microglia were induced with PA (100 μM) and pharmacological treatment with ART (0, 0.1, 0.5, 1.0 and 2.5 μM), STAU (a pan-PKC inhibitor, 1.0 μM), or 2BP (a protein palmitoylation inhibitor, 1.0 μM) was carried out in the meantime.

For hepatic treatment, LO2, HepG2 cells or primary hepatocytes were cultured on coverslips or 6-well plates and induced with PA (100 μM) or OA (300 μM) saponified by 0.1% NaOH with the treatment of 0.5% BSA for 24 h. Then, the cells were treated with ART (0.1, 0.5, 1.0 and 2.5 μM) or *l*-THY (12.5, 25, 50 and 100 μM) for another 24 h.

For neurons and microglia co-culture, BV-2 cells were treated with PA (100 μM) and ART (0, 0.1, 0.5, 1.0 and 2.5 μM) for 2 h. Cell culture supernatants were abandoned and changed to serum free SFM neurobasal medium containing B-27 supplement for 24 h incubation. Primary neurons were treated with the BV-2 cell conditional medium (CM) in the absence or the presence of ART for another 24 h. Then, further experiments were carried out.

### Serum biochemical analysis

Blood samples from each experimental group were taken from the eye socket after 12 h of fasting under anaesthesia and serum was collected for biochemical analysis. The concentrations of TC, TG, HDL-C, ALT, and AST in serum were measured by BS180 automated clinical biochemistry analyzer (Mindray, Shenzhen, Guangdong, China) according to the manufacturer's instructions. Non-HDL-C was calculated as follows: non-HDL-C = TC-HDL-C.

### Oil red O staining

Liver tissues were frozen in optim alcutting tem perature (OCT) and sectioned on slides. Tissue sections (4 μm) were rinsed immediately in distilled water for several seconds, and then placed in isopropanol for 30 s. Tissue sections were incubated in 0.3% oil red O isopropanol-water (v/v = 3:2) for 30 min and stained with 5% haematoxylin solution for 5 min after being rinsed in distilled water. Glycerin jelly was used to mount with tissue sections, and images were observed by NanoZoomer-SQ Digital slide scanner (Hamamatsu Photonics, Hamamatsu, Japan). Quantification for the area of hepatic lipid accumulation was carried out by image processing software pro plus 6.0 (Media Cybernetics, Shanghai, China).

### Histology and immunohistochemistry

For H&E staining, tissue sections (4 μm) were then dehydrated with different concentrations of xylol and ethanol, followed by incubation with 5% haematoxylin solution for 15 min. After washes, the sections were stained in 0.1% HCl-ethanol for 30 s and then reacted with eosin solution for 2 min.

For immunohistochemistry, tissue sections (4 μm) were reacted with 3% H_2_O_2_ in methanol for 15 min, and then incubated with primary antibodies against Iba1 (1:100) and TRH (1:100) at 4 °C overnight. After washing with PBS, secondary antibody was performed for staining with tissue sections at room temperature for 1 h. DAB substrate was used to incubate with the sections for visualization. Images were observed by NanoZoomer-SQ Digital slide scanner (Hamamatsu Photonics, Hamamatsu, Japan), and quantification for the area of hepatic lipid damage was carried out by image processing software pro plus 6.0. The number of positive cells were analyzed by Image J software. Neuron J plugin in Image J software was applied to trace branching points and calculate dendritic length. Dendritic intersections from cell bodies were measured by Sholl analysis. Branching points and dendritic lengths were detected from primary dendrites to secondary dendrites.

### Quantitative real time-polymerase chain reaction (RT-PCR)

Total RNA was isolated by MolPure^®^ Cell/Tissue Total RNA Kit (#19221ES50, TEASEN, Shanghai, China) from cells or tissues, and was quantified in a NanoDrop 3000 (ThermoFisher, Waltham, MA, USA). First-strand cDNA synthesis was carried out using Hifair^®^ II 1st Strand cDNA Synthesis Kit (#11141ES60, TEASEN, Shanghai, China). Quantitative PCR was performed with Hieff^®^ qPCR SYBR Green Master Mix (#11202ES08, TEASEN, Shanghai, China) using Agilent Technologies Stratagene Mx3005P System (Santa Clara, CA, USA). PCR conditions were as follow: 50 °C for 2 min, 95 °C for 10 min, and 40 cycles of 95 °C for 30 s, 60 °C for 30 s, and 72 °C for 30 s. The sequences of qPCR primers (Generay, Shanghai, China) applied for quantitative PCR in this study were shown in table S4.

### Transport experiments

Caco-2 cells were seeded in 12-well polyester insert plates (3 μm of pore size) and cultured until TEER voltage (Epithelial Volt/Ohm Meter, WPI, Sarasota, FL, USA) was measured to ensure membrane integrity prior (>650 O). Caco-2 cell monolayers were washed twice with 37 °C pre-warmed HBSS. Then, the cells were treated with SA (80 μM), PA (80 μM), OA (20 μM), or CHO (10 μM) in the presence of ART (0, and 2.5 μM). The system was incubated at 37 °C under 95% absolute humidity in a 5% CO_2_ incubator. Samples were collected from the basolateral side at 0, 20, 40, 60, 80, 100, and 120 min.

The transports of OA, PA, SA, and CHO were quantitatively detected with an UPLC/Qtrap-MS system, conducted on a Waters ACQUITY UPLC system connected online with an ABSciex 4500 Qtrap massspectrometer via an electrospray ionization (ESI) interface (Foster City, CA, USA). The ion source parameters were maintained as follows: positive or negative ion mode; ion spray voltage, 5500 V or -4500 V; source temperature, 500 ℃, 90 V; curtain gas (CUR), 35 psi; ion source gas 1 (GS1), 50 psi; ion source gas 2 (GS2), 50 psi. The declustering potential (DP) and collision energy (CE) of OA, PA, SA, and CHO were shown in table S5. Chromatographic separations were conducted on an ACQUITY UPLC HSS T3 column (2.1×100 mm, 1.8 μm). OA, PA, and SA were detected with mobile phase consisting of 0.1% acetic acid water (A) and acetonitrile (B), while CHO was detected with 0.1% formic water (A) and acetonitrile (B), in gradient as follows: 0-6 min, 10-100% B; 6-8 min, 100% B. The flow rate was 0.4 mL/min.

### Nile red staining

After treatment, cells were washed twice with PBS and fixed with 4% paraformaldehyde at room temperature for 30 min. Then, cells were stained with 1 μg/mL Nile Red for 10 min at 37 °C and washed three times with PBS. Images were recorded with a Zeiss LSM880 (Carl Zeiss, Oberkochen, Germany) with Airyscan microscope and captured by ZEN2 software. Relative fluorescence intensity was analyzed by Image J software.

### TSH, FT4, FT3, adrenaline, insulin, and glucagon measurement

The concentrations of TSH, FT4, FT3, adrenaline, insulin, and glucagon were measured with ELISA kits (TSH assay kit, #H087, FT4 assay kit, #H225, FT3 assay kit, #H224, adrenaline assay kit, #H208, insulin assay kit, #H203, glucagon assay kit, #H183, JianchengBio, Nanjing, Jiangsu, China). Briefly, 50 μL mouse serum was reacted with 50 μL biotin-conjugate in miceowell plates at 37 °C. After 30 min incubation, the miceowell plates were washed for five times and incubated with 50 μL streptavidin-HRP for 30 min at 37 °C. 100 μL substrate solutions (substrate solution A: substrate solution B=1:1, v/v) were performed to carry out enzymatic reaction for 10 min. 50 μL stop solutions were added to stop the reaction. The absorbance was determined under 450 nm.

### Immunofluorescence

Tissue sections (4 μm) were permeabilized with 0.5% Triton X-100 for 5 min and blocked at room temperature with 5% bovine serum albumin. Primary antibodies against mouse Iba1 (1:100), TRH (1:100), GFAP (1:100), PKCδ(1:100), THRβ (1:100), TNF-α (1:100) and IL-6 (1:100) were used for reaction with tissue sections overnight at 4 °C, followed by a 60 min incubation with Alexa Fluor 488 goat anti-rabbit or Alexa Fluor 594 goat anti-mouse secondary antibodies at room temperature. After being washed for three times, tissue sections were stained with DAPI at a concentration of 1:10000 in PBS for 5 min. Images were observed by NanoZoomer-SQ Digital slide scanner (Hamamatsu Photonics, Hamamatsu, Japan) with excitation/emission wavelengths of 495 nm/520 nm for Alexa Fluor 488, 590 nm/617 nm for Alexa Fluor 594, and 360 nm/450 nm for DAPI. Relative fluorescence intensity was analyzed by Image J software.

### Cell viability analysis

After treatment, cell culture supernatants were abandoned and 3-(4, 5-dimethyl thiazol-2-yl)-2, 5-diphenyl tetrazolium bromide (MTT) solution (0.5 mg/mL) was added in 48-well plates. The system was kept at 37 °C in a 5% CO_2_ humidified incubator for 4 h. Cell culture supernatants were abandoned again and formazan crystal was dissolved in DMSO with shaking for 15 min. The absorbance was determined under 570 nm.

### NO, TNF-α and IL-6 measurement

After treatment, cell supernatants were collected to analyze NO (nitric oxide analysis kit, #A013, JianchengBio, Nanjing, Jiangsu, China), TNF-α (mouse TNF-α ELISA kit, #EM008, ExCellBio, Shanghai, China) or IL-6 (mouse IL-6 ELISA kit, #EM004, ExCellBio, Shanghai, China) levels.

For NO analysis, 200 μL 200 μL Griess reagent (1% sulfanilamide/ 0.1% naphthylethylene diamine dihydrochloride/ 2% phosphoric acid) was added to 100 μL cell supernatants followed by vortex mixing and standing for 10 min. The absorbance was measured under 570 nm.

For TNF-α and IL-6 measurement, 100 μL cell supernatants were transferred to miceowell plates and incubated with 50 μL biotin-conjugate for 2 h. Then, the miceowell plates were washed for five times and 100 μL streptavidin-HRP were added for 1 h incubation. After being washed, the miceowell plates were reacted with 100 μL substrate solutions for 10 min. 100 μL stop solutions were used to stop the color change reaction and the absorbance was determined under 450 nm.

### Thermal proteome profiling (TPP)

Heavy L-Arginine and L-Lysine (^13^C/^15^N) or light L-Arginine and L-Lysine (^12^C/^14^N) were dissolved in SILAC medium (#CM00001, MACGENE, Beijing, China) supplemented with 10% dialyzed FBS (#04-011-1A, BioRuler, Beijing, China), 100 U/mL penicillin, and 100 μg/mL streptomycin to culture BV-2 cells for 6 generations. Then, light amino acids-labeled cells were incubated with 2.5 μM ART for 2 h, while heavy amino acids-labeled cells were incubated with DMSO. After treatment, the labeled cells were heated at 45 °C for 3 min, and then cooled on ice quickly. Kinase buffer was added to maintain kinase activity (#9802, Cell Signaling Technology, Beverly, MA, USA), and liquid nitrogen was used to freeze-thaw the labeled cells twice. BCA protein assay kit (#DQ111, TransGen, Beijing, China) was performed to prepare protein solutions of equal concentration. Subsequently, light amino acids-labeled protein solutions were mixed with heavy amino acids-labeled protein solutions in equal proportion.

Protein solutions were digested with trypsin and the extracted peptides were analyzed on an EASY-nLC II HPLC system connected to a LTQ-Orbitrap velos pro mass spectrometer (ThermoFisher, Waltham, MA, USA) at a constant flow rate of 300 nL/min with a following gradient: buffer B from 2% to 40% in 70 min; from 40% to 95% in 5 min; 95% in 20 min (buffer A, 0.1% formic acid in water, v/v; buffer B, 0.1% formic acid in acetonitrile, v/v). The eluted peptides were ionized and sprayed into the mass spectrometer through a nano-electrospray ion source in a data dependent mode. The signals were obtained with a full scan MS spectra (mass-to-charge ratio range, 350-2000), and analyzed in an Orbitrap analyzer with a resolution of 60,000. The top 15 abundant precursor ions were selected from each MS and fragmented by higher-energy collisional dissociation with a normalized collision energy of 35%. MS data were analyzed via Proteome Discoverer (1.4) software combined with the SEQUEST search engine (ThermoFisher, Waltham, MA, USA).

### Transcriptomics, bioinformatics and connectivitymap analysis

After treatment for 6 h, cells were collected and total RNA was extracted by the RNAprep Pure Cell/Bacteria Kit (#DP430-H, Tiangen Biotech, Beijing, China). Then, transcriptomics analysis was carried out by Novogene (Beijing, China). Superscript II reverse transcriptase (Invitrogen, Carlsbad, CA, USA) and random hexamer primers were used to synthesize double-stranded complementary DNAs. NEBNext Ultra RNA Library Prep Kit for Illumina was performed to generate and sequence mRNA library. The different gene expressions between any two groups were obtained through the DESeq R package (1.18.0). The genes of *P* values < 0.05 and a log2 (fold change) < -1 between ART group and PA group were selected. KEGG pathway enrichment analysis was performed to determine the signaling pathway affected by ART through comparing ART group with PA group. Signaling pathways highly related with inflammation were selected using Cytoscape v3.6.0 under Mus musculus genome information. Meantime, the top 100 genes significantly influenced by ART were selected according to data of transcriptomics analysis 56. The targets of ART were predicted using ConnectivityMap analysis and top 20 functions with significant positive connectivity scores were selected.

### Pull-down assay

Avidin agaroses were washed by PBS twice and incubated with biotin-ART (Extended Data Reaction) for 2 h. Whole cell or tissue proteins were isolated by ice-cold NP-40 buffer containing 1% protease inhibitors. Then, protein solutions were incubated with biotin-ART-binding avidin agaroses (agarose-ART) or control avidin agaroses in the presence or absence of free ART (0, 10, 50 and 200 folds) for competition. Agaroses were washed six times by washing buffer (50 mM Tris, 5 mM EDTA, 100 mM NaCl, 1 mM DTT, 0.01% Triton X-100). Protein loading buffer was used to collect the captured proteins. Western blotting was performed to analyze PKCα, PKCδ, PKCζ, and PKCμ proteins.

### Cellular thermal shift Assay (CETSA)

After treatment with or without ART, BV-2 cells were heated at gradually rising temperatures (37 to 61 °C) for 3 min, and then cooled on ice 57. Subsequently, kinase buffer was added, and cells were freeze-thawed three times using liquid nitrogen. Western blotting was performed to analyze PKCα, PKCδ, PKCζ, and PKCμ proteins.

### Drug affinity responsive target stability (DARTS)

Whole cell proteins were isolated by ice-cold NP-40 buffer (#KGP705, KeChuang Biotechnology, Suzhou, Jiangsu, China) containing 1% protease inhibitors (#P10015, Leagene, Beijing, China) 58. TNC buffer (50 mM Tris·Cl, pH 8.0, 50 mM NaCl, 10 mM CaCl_2_) was used to dilute protein solutions. Then, protein solutions were treated with ART (0, 0.6, 1.2 and 2.5 μM) for 1 h at room temperature. Subsequently, pronase was used to react with protein solutions for another 15 min at room temperature. Protein loading buffer was added to stop the reactions and western blotting was performed to analyze PKCα, PKCδ, PKCζ, and PKCμ proteins.

### Acyl-biotinyl exchange for palmitoylated proteins analysis

Protein solutions were prepared by ice-cold NP-40 buffer containing 1% protease inhibitors and collected by centrifuging under 14000 rpm for 20 min. N-ethylmaleimide (NEM, 20 mM) was added to protein solutions of equal concentration, and the samples were incubated at 4 °C for 2 h. Methanol precipitation was performed three times and the pellet was resuspended in solubilisation buffer (4% SDS, 50 mM Tris·HCl, 5 mM EDTA, pH 7.4) for incubation at room temperature for 5 min. Hydroxylamine (HA, pH 7.4, 25% final concentration) and EZ-link® N-[6-(biotinamido) hexyl]-3′ -(2′ -pyridyldithio)propionamide (biotin-HPDP) were added to incubate with protein solutions at 4 °C overnight. Avidin agaroses were washed by PBS twice and interacted with the mixture. SDS loading buffer was used to collect the remained proteins and western blotting was performed to analyze the captured PKCδ.

### Small interfering RNA (siRNA) or plasmid transfection

BV-2, LO2 or HepG2 were transfected with siRNA specifically targeting *Prkcd* or *Zdhhc5* using Lipofectamine RNAi MAX (#13778150, Invitrogen, Carlsbad, CA, USA) according to the manufacturer's instructions. The sequence of the antisense siRNA targeting human *Prkcd* was as follows (5'-3'): GCUGGACAAUGUGAUGCUATT. The sequence of the antisense siRNA targeting mouse *Prkcd* was as follows (5'-3'): GGGAUUAAAGUGUGAAGAUTT. The sequence of the antisense siRNA targeting mouse *Zdhhc5* was as follows (5'-3'): GAAAGAGAAGACAAUUGUATT. After transfection for 24 h in normal DMEM, cells were treated as described before for further analysis. For plasmid transfection, HEK293T or BV-2 cells were transfected with 24 μg indicated plasmid (Generay, Shanghai, China) using Lipofectamine 2000 transfection reagent in Opti-MEM (#31985-070, Invitrogen, Carlsbad, CA, USA). After 6 h incubation, the medium was changed to complete DMEM, and the cells were incubated for 48 h before experiments.

### Co-immunoprecipitation (Co-IP) analysis

HEK293T cells were transfected with desired plasmids and then lysed with NP-40 lysis buffer containing 1% protease inhibitors with shaking for 30 min. Cell homogenates were centrifuged under 14000 rpm for 20 min, and a total of 500 μL protein solution was incubated with 25 μL HA-tag beads (#C29F4, Invitrogen, Carlsbad, CA, USA) overnight at 4 °C. Finally, the beads were washed 5-6 times with cold washing buffer (50 mM Tris, 5 mM EDTA, 100 mM NaCl, 1 mM DTT, 0.01% Triton X-100) and collected in SDS loading buffer for western blotting.

Cultured LO2 cells were lysed with lysis buffer NP-40 containing 1% protease inhibitors with constant agitation and then centrifuged. A total of 500 μL protein solution was incubated with 25 μL Protein A/G agarose beads (#HYK0202, MedChemExpress, Shanghai, China) and the indicated antibody with rocking overnight at 4 °C. Finally, the beads were washed 5-6 times with cold washing buffer (50 mM Tris, 5 mM EDTA, 100 mM NaCl, 1 mM DTT, 0.01% Triton X-100) and collected in SDS loading buffer for western blotting.

### Western blotting (WB)

After treatment, whole cell proteins were extracted by ice-cold NP-40 buffer containing 1% protease inhibitors with shaking for 30 min, and collected by centrifuging under 14000 rpm for 20 min. Protein solutions were separated on 10-15% SDS-PAGE and transferred to PVDF membranes. Subsequently, 5% skim milk was performed to block PVDF membranes at 25 °C for 30 min. Then, membranes were incubated with different primary antibodies at 4 °C for 10 h and second antibody at 25 °C for 1 h. Finally, membranes were developed with SuperSignal West Femto Maximum Sensitivity Substrate and imaged by Tanon 5200 Imaging Analysis System (Shanghai, China). Related densitometry analysis was determined by Image J software.

### UPLC-MS/MS for measuring ART concentration in hypothalamus

C57BL/6J mice were treated with 20 mg/kg by oral administration for 4 h. After perfusion with sodium chloride injection, hypothalamic homogenate (10 mg/mL) was prepared in 50% methanol-water and centrifuged at 14000 rpm for 10 min. 400 ng/mL artesunate was added and 1 μL supernatant was injected for UPLC-MS/MS analysis (Foster City, CA, USA). The ion source parameters were maintained as follows: positive or negative ion mode; ion spray voltage, 5500 V or -4500 V; source temperature, 500 °C, 90 V; curtain gas (CUR), 35 psi; ion source gas 1 (GS1), 50 psi; ion source gas 2 (GS2), 50 psi. The DP and CE of ART and artesunate were shown in Table S5. Chromatographic separations were conducted on an ACQUITY UPLC HSS T3 column (2.1×100 mm, 1.8 μm). ART and artesunate were detected with mobile phase consisting of 0.1% ammonium acetate water (A) and acetonitrile (B) in gradient as follows: 0-6 min, 20-100 % B; 6-8 min, 100 % B. The flow rate was 0.4 mL/min.

### Single-cell RNA sequencing

Brain tissues were stored in the sCelLive^TM^ Tissue Preservation Solution on ice after surgery and washed with HBSS for three times. Then, the specimens were minced into small pieces and digested with sCelLiveTM Tissue Dissociation Solution (Singleron) by Singleron PythoN^TM^ Tissue Dissociation System (Singleron, Suzhou, China) at 37 °C for 15 min. The cell suspension was collected and filtered through a 40-micron sterile strainer. Afterwards, the GEXSCOPE® red blood cell lysis buffer was added to remove red blood cells. The mixture was then centrifuged at 1000 rpm for 5 min to remove supernatant and suspended softly with PBS.

Single-cell suspensions (2×10^5^ cells/mL) prepared with PBS were loaded onto microwell chip using the Singleron Matrix^®^ Single Cell Processing System (Singleron, Suzhou, China). Barcoding Beads were collected from the microwell chip, followed by reverse transcription to obtain cDNA. The amplified cDNA was fragmented and ligated with sequencing adapters and single-cell RNA sequencing libraries were constructed according to the GEXSCOPE^®^ Single Cell RNA Library Kits. Individual libraries were diluted to 4 nM and then sequenced on Illumina novaseq 6000 with 150 bp paired-end reads.

Raw reads from single-cell RNA sequencing were processed to generate gene expression matrixes using CeleScope (https://github.com/singleron-RD/CeleScope) v1.9.0 pipeline. Briefly, raw reads were first processed with CeleScope to remove low quality reads and trim poly-A tail and adapter sequences. After extracting cell barcode and UMI, STAR v2.6.1a were used for mapping reads to the reference genome GRCh38. UMI and gene counts of every cell were acquired with featureCounts v2.0.1 software, which generated expression matrix files for subsequent analysis 59.

### Statistical analysis

GraphPad Prism 6.0 software was determined to carry out statistical analysis *via* one-way analysis of variance (ANOVA) without correcting for multiple comparisons. Student's *t*-test was evaluated by comparing the mean of each column with the mean of every other column. All experiments were performed at least three times with triplicate. All data were expressed as means ± S.D. *p* value < 0.05 was considered to be significant.

## Supplementary Material

Supplementary figures and tables, information.
