# Supplementary material for: Palmitoylation of PKCδ by ZDHHC5 in hypothalamic microglia presents as a therapeutic target for fatty liver disease
Source: Theranostics. 2024 Jan 1;14(3):988–1009. doi: 10.7150/thno.89602 (PMC10797291; doi:10.7150/thno.89602)
Supplement: Supplementary file 1 — Supplementary figures and tables, information. [file thnov14p0988s1.pdf]

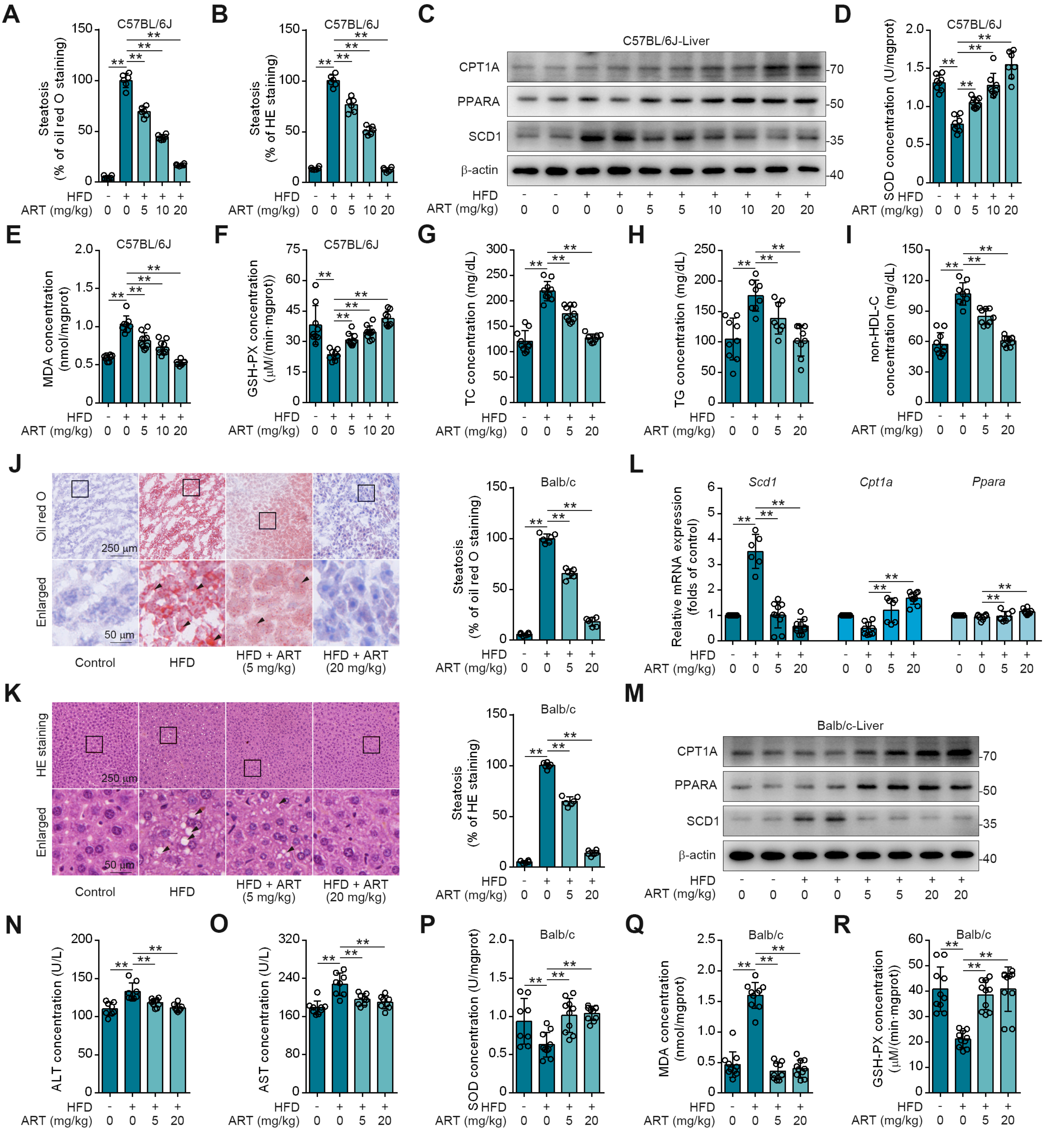
A
C57BL/6J
Steatosis (% of oil red O staining)
HFD - + + + +
ART (mg/kg) 0 0 5 10 20
B
C57BL/6J
Steatosis (% of HE staining)
HFD - + + + +
ART (mg/kg) 0 0 5 10 20
C
C57BL/6J-Liver
CPT1A
PPARA
SCD1
β-actin
-70
-50
-35
-40
HFD - - + + + + + + + +
ART (mg/kg) 0 0 0 0 5 5 10 10 20 20
D
C57BL/6J
SOD concentration (U/mgprot)
HFD - + + + +
ART (mg/kg) 0 0 5 10 20
E
C57BL/6J
MDA concentration (nmol/mgprot)
HFD - + + + +
ART (mg/kg) 0 0 5 10 20
F
C57BL/6J
GSH-PX concentration (μM/(min·mgprot))
HFD - + + + +
ART (mg/kg) 0 0 5 10 20
G
TC concentration (mg/dL)
HFD - + + +
ART (mg/kg) 0 0 5 20
H
TG concentration (mg/dL)
HFD - + + +
ART (mg/kg) 0 0 5 20
I
non-HDL-C concentration (mg/dL)
HFD - + + +
ART (mg/kg) 0 0 5 20
J
Oil red O
Enlarged
250 μm
50 μm
Control
HFD
HFD + ART (5 mg/kg)
HFD + ART (20 mg/kg)
Balb/c
Steatosis (% of oil red O staining)
HFD - + + +
ART (mg/kg) 0 0 5 20
L
Scd1
Cpt1a
Ppara
Relative mRNA expression (folds of control)
HFD - + + + - + + + - + + +
ART (mg/kg) 0 0 5 20 0 0 5 20 0 0 5 20
K
HE staining
Enlarged
250 μm
50 μm
Control
HFD
HFD + ART (5 mg/kg)
HFD + ART (20 mg/kg)
Balb/c
Steatosis (% of HE staining)
HFD - + + +
ART (mg/kg) 0 0 5 20
M
Balb/c-Liver
CPT1A
PPARA
SCD1
β-actin
-70
-50
-35
-40
HFD - - + + + + + +
ART (mg/kg) 0 0 0 0 5 5 20 20
N
ALT concentration (U/L)
HFD - + + +
ART (mg/kg) 0 0 5 20
O
AST concentration (U/L)
HFD - + + +
ART (mg/kg) 0 0 5 20
P
Balb/c
SOD concentration (U/mgprot)
HFD - + + +
ART (mg/kg) 0 0 5 20
Q
Balb/c
MDA concentration (nmol/mgprot)
HFD - + + +
ART (mg/kg) 0 0 5 20
R
Balb/c
GSH-PX concentration (μM/(min·mgprot))
HFD - + + +
ART (mg/kg) 0 0 5 20

Supporting Information Figure S1 ART inhibits HFD-fed hepatic lipid metabolism disorder in C57BL/6J and Balb/c mice.

(A) Quantification for the area of hepatic lipid accumulation was relative to HFD group in HFD-fed C57BL/6J mice treated with ART (n = 6 visual fields each group).

(B) Quantification for the area of hepatic lipid damage was relative to HFD group in HFD-fed C57BL/6J mice treated with ART (n = 6 visual fields each group).

(C) Immunoblots of CPT1A, PPARA and SCD1 from liver in HFD-fed C57BL/6J mice treated with ART.

(D–F) SOD (D), MDA (E), and GSH-PX (F) assay of liver in HFD-fed C57BL/6J mice treated with ART (n = 7–10 mice each group).

(G–I) TC (G), TG (H), and non-HDL-C (I) assay of HFD-fed Balb/c mice treated with ART in serum (n = 6–10 mice each group).

(J) Oil red O staining of hepatic lipid accumulation in HFD-fed Balb/c mice treated with ART; quantification for the area of hepatic lipid accumulation was relative to HFD group (n = 6 visual fields each group).

(K) HE staining of hepatic lipid damage in HFD-fed Balb/c mice treated with ART; quantification for the area of hepatic lipid damage was relative to HFD group (n = 6 visual fields each group).

(L) *Scd1*, *Cpt1a* and *Ppara* mRNA levels of HFD-fed Balb/c mice treated with ART (n = 6–10 mice each group).

(M) Immunoblots of CPT1A, PPARA and SCD1 from liver in HFD-fed Balb/c mice treated with ART.

(N–O) ALT (N), and AST (O) assay of HFD-fed Balb/c mice treated with ART in serum (n = 8–9 mice each group).

(P–R) SOD (P), MDA (Q), and GSH-PX (R) assay of liver in HFD-fed Balb/c mice treated with ART (n = 8–10 mice each group).

Statistical comparisons were analyzed by one-way ANOVA analysis followed by Student's t-test. Data are presented as mean ± SD. $^{**}p < 0.01$.

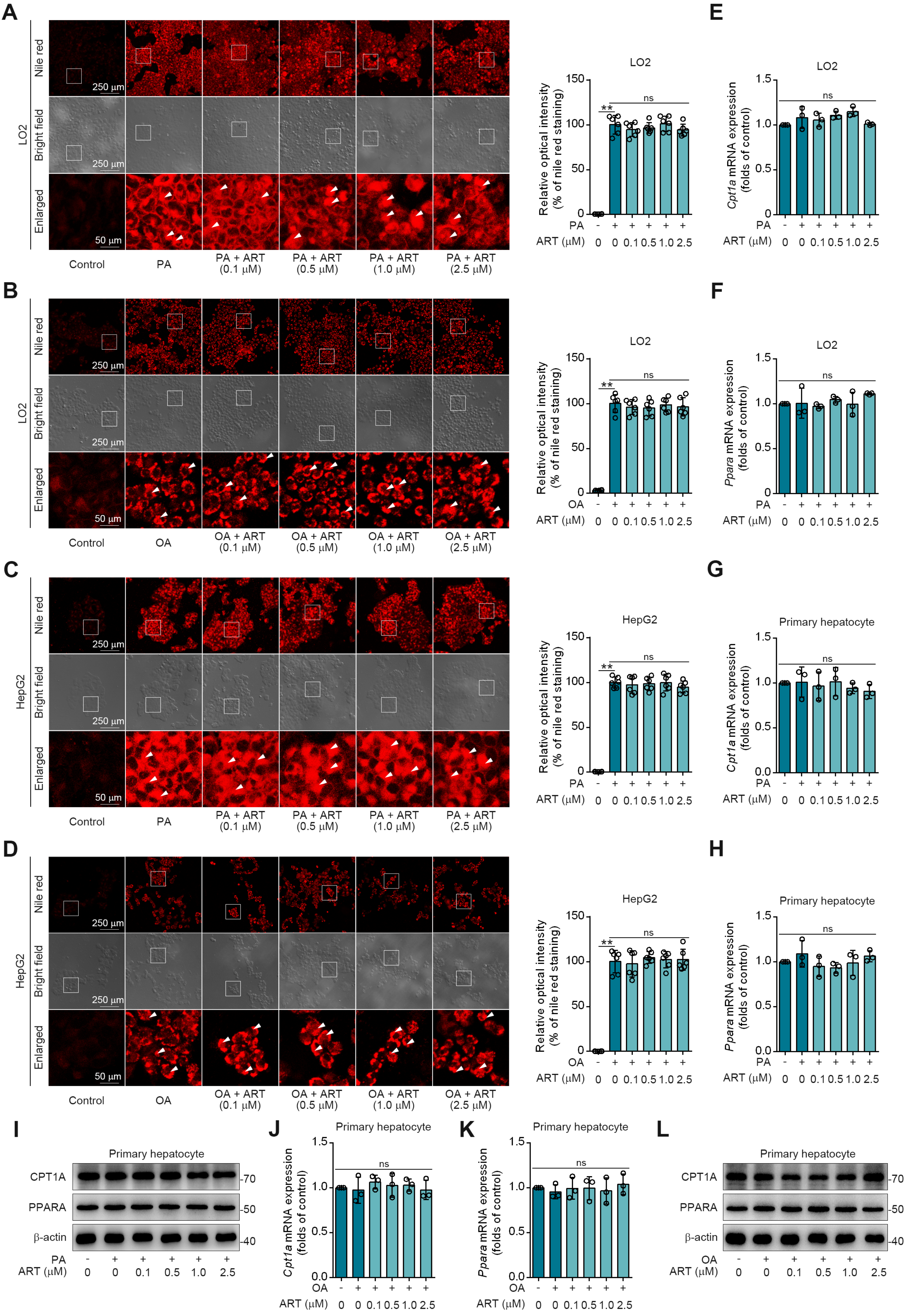

A
B
C
D
E
F
G
H
I
J
K
L
LO2
HepG2
Nile red
Bright field
Enlarged
250 μm
50 μm
Control
PA
OA
PA + ART (0.1 μM)
PA + ART (0.5 μM)
PA + ART (1.0 μM)
PA + ART (2.5 μM)
OA + ART (0.1 μM)
OA + ART (0.5 μM)
OA + ART (1.0 μM)
OA + ART (2.5 μM)
Relative optical intensity (% of nile red staining)
Cpt1a mRNA expression (folds of control)
Ppara mRNA expression (folds of control)
Primary hepatocyte
ART (μM)
0 0 0.1 0.5 1.0 2.5
ns
**
CPT1A
PPARA
β-actin
-70
-50
-40

Supporting Information Figure S2 ART has no effect on hepatocyte lipid accumulation.

(A) Nile red staining of hepatocyte lipid accumulation in PA-induced LO2 cells treated with ART; quantification for immunofluorescence intensity was relative to PA group (n = 6 visual fields each group).

(B) Nile red staining of hepatocyte lipid accumulation in OA-induced LO2 cells treated with ART; quantification for immunofluorescence intensity was relative to OA group (n = 6 visual fields each group).

(C) Nile red staining of hepatocyte lipid accumulation in PA-induced HepG2 cells treated with ART; quantification for immunofluorescence intensity was relative to PA group (n = 6 visual fields each group).

(D) Nile red staining of hepatocyte lipid accumulation in OA-induced HepG2 cells treated with ART; quantification for immunofluorescence intensity was relative to OA group (n = 6 visual fields each group).

(E–F) *Cpt1a* (E) and *Ppara* (F) mRNA levels of PA-induced LO2 cells treated with ART (n = 3 each group).

(G–H) *Cpt1a* (G) and *Ppara* (H) mRNA levels of PA-induced primary hepatocytes treated with ART (n = 3 each group).

(I) Immunoblots of CPT1A and PPARA of PA-induced primary hepatocytes treated with ART.

(J–K) *Cpt1a* (J) and *Ppara* (K) mRNA levels of OA-induced primary hepatocytes treated with ART (n = 3 each group).

(L) Immunoblots of CPT1A and PPARA of OA-induced primary hepatocytes treated with ART.

Statistical comparisons were analyzed by one-way ANOVA analysis followed by Student's t-test. Data are presented as mean ± SD. $^{**}p < 0.01$.

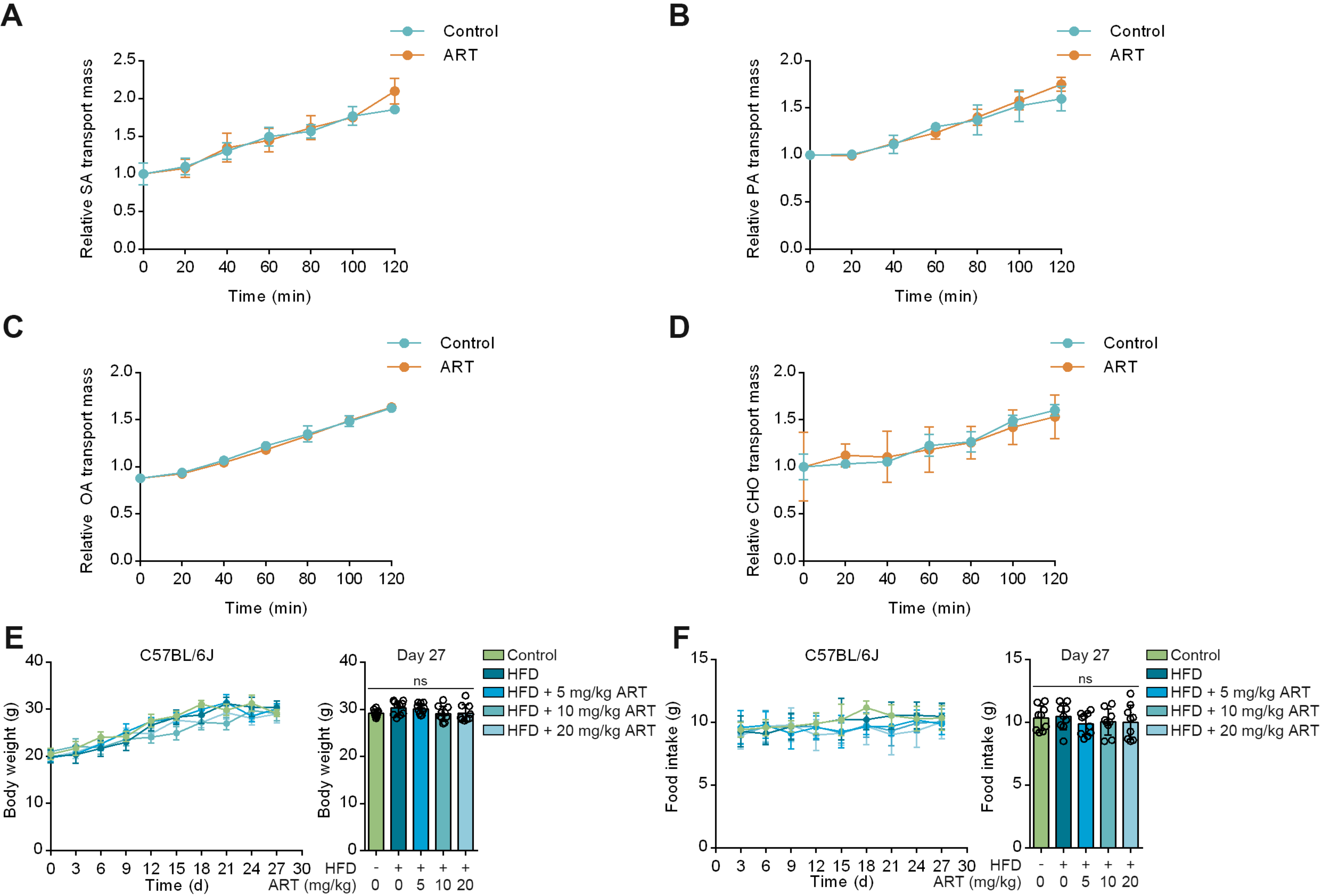

A
B
C
D
E
F
Control
ART
Relative SA transport mass
Relative PA transport mass
Relative OA transport mass
Relative CHO transport mass
Time (min)
C57BL/6J
Day 27
ns
Body weight (g)
Food intake (g)
Time (d)
HFD
ART (mg/kg)
HFD + 5 mg/kg ART
HFD + 10 mg/kg ART
HFD + 20 mg/kg ART

Supporting Information Figure S3 ART does not interfere with intestinal absorption.

(A–D) Relative SA (A), PA (B), OA (C), and CHO (D) transport mass from AP to BL side in Caco-2 cells treated with ART (n = 3 mice each group).

(E–F) Body weight (E), and food intake (F) of HFD-fed C57BL/6J mice treated with ART (n = 9–10 mice each group).

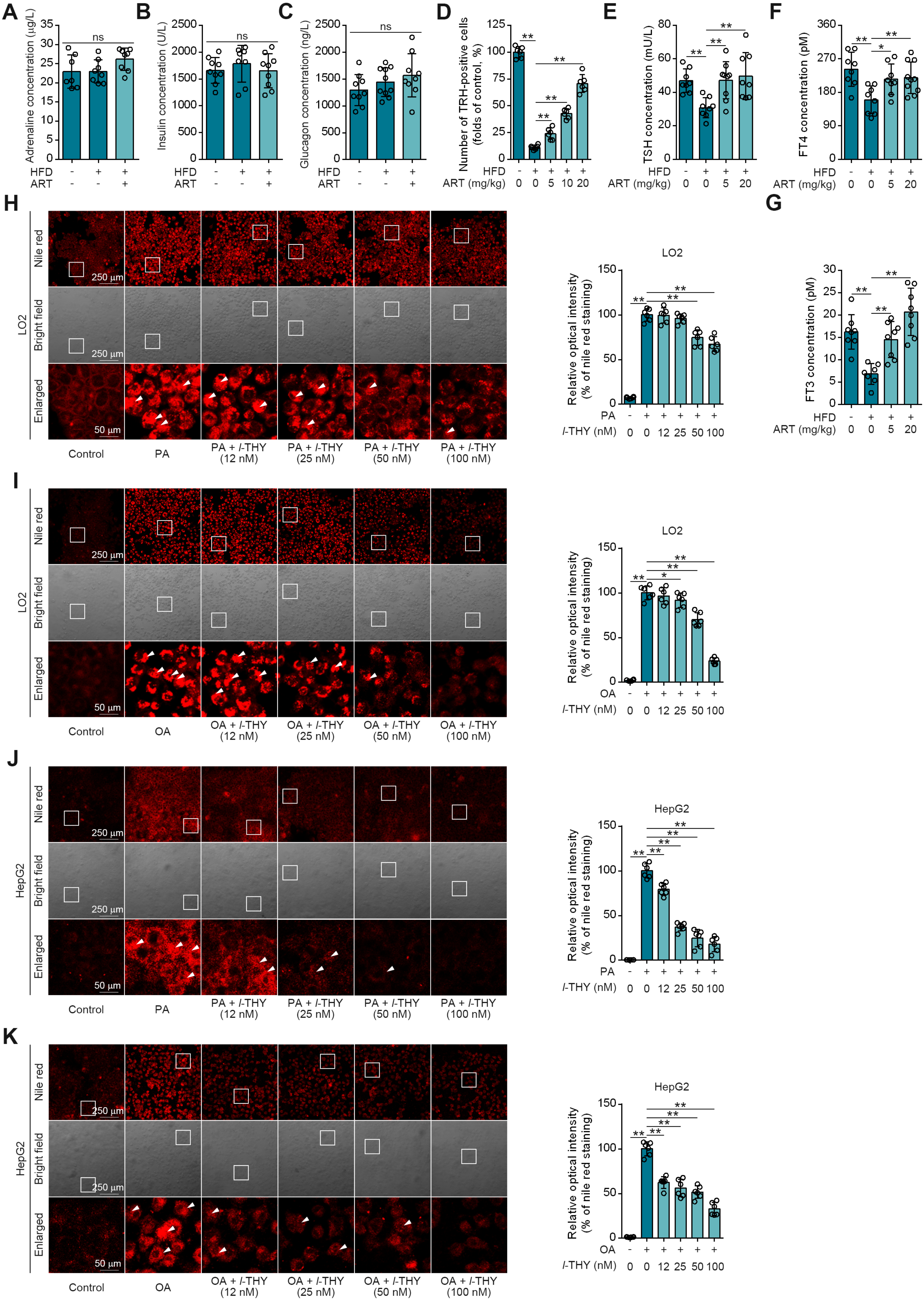

A
Adrenaline concentration (μg/L)
ns
HFD - + +
ART - - +
B
Insulin concentration (U/L)
ns
HFD - + +
ART - - +
C
Glucagon concentration (ng/L)
ns
HFD - + +
ART - - +
D
Number of TRH-positive cells (folds of control, %)
HFD - + + + +
ART (mg/kg) 0 0 5 10 20
E
TSH concentration (mU/L)
HFD - + + +
ART (mg/kg) 0 0 5 20
F
FT4 concentration (pM)
HFD - + + +
ART (mg/kg) 0 0 5 20
G
FT3 concentration (pM)
HFD - + + +
ART (mg/kg) 0 0 5 20
H
LO2
Nile red
Bright field
Enlarged
250 μm
50 μm
Control
PA
PA + l-THY (12 nM)
PA + l-THY (25 nM)
PA + l-THY (50 nM)
PA + l-THY (100 nM)
Relative optical intensity (% of nile red staining)
PA - + + + + +
l-THY (nM) 0 0 12 25 50 100
I
LO2
Control
OA
OA + l-THY (12 nM)
OA + l-THY (25 nM)
OA + l-THY (50 nM)
OA + l-THY (100 nM)
OA - + + + + +
l-THY (nM) 0 0 12 25 50 100
J
HepG2
Control
PA
PA + l-THY (12 nM)
PA + l-THY (25 nM)
PA + l-THY (50 nM)
PA + l-THY (100 nM)
PA - + + + + +
l-THY (nM) 0 0 12 25 50 100
K
HepG2
Control
OA
OA + l-THY (12 nM)
OA + l-THY (25 nM)
OA + l-THY (50 nM)
OA + l-THY (100 nM)
OA - + + + + +
l-THY (nM) 0 0 12 25 50 100

Supporting Information Figure S4 ART suppressed hepatocyte lipid accumulation by promoting TH release.

(A–C) Adrenaline (A), insulin (B), and glucagon (C) assay of HFD-fed C57BL/6J mice treated with ART in serum (n = 7–10 mice each group).

(D) Quantification for the number of TRH-positive cells was relative to control group in HFD-fed C57BL/6J mice treated with ART (n = 6 visual fields each group).

(E–G) TSH (E), FT4 (F), and FT3 (G) assay of HFD-fed Balb/c mice treated with ART in serum (n = 7–10 mice each group).

(H) Nile red staining of hepatocyte lipid accumulation in PA-induced LO2 cells treated with *l*-THY; quantification for immunofluorescence intensity was relative to PA group (n = 6 visual fields each group).

(I) Nile red staining of hepatocyte lipid accumulation in OA-induced LO2 cells treated with *l*-THY; quantification for immunofluorescence intensity was relative to OA group (n = 6 visual fields each group).

(J) Nile red staining of hepatocyte lipid accumulation in PA-induced HepG2 cells treated with *l*-THY; quantification for immunofluorescence intensity was relative to PA group (n = 6 visual fields each group).

(K) Nile red staining of hepatocyte lipid accumulation in OA-induced HepG2 cells treated with *l*-THY; quantification for immunofluorescence intensity was relative to OA group (n = 6 visual fields each group).

Statistical comparisons were analyzed by one-way ANOVA analysis followed by Student’s t-test. Data are presented as mean ± SD. $^{*}p < 0.05$, $^{**}p < 0.01$.

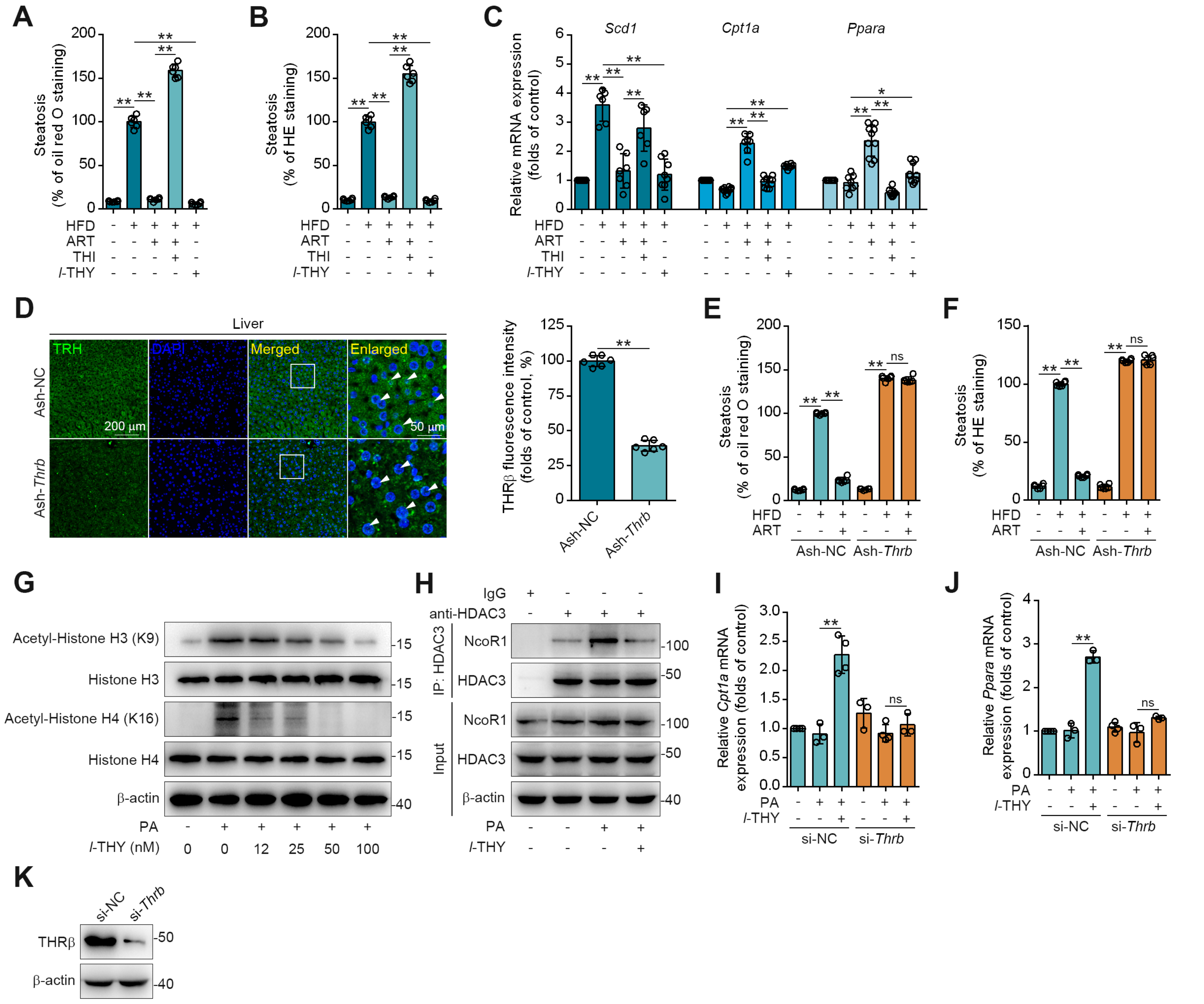
A
Steatosis (% of oil red O staining)
HFD
ART
THI
l-THY
B
Steatosis (% of HE staining)
C
Scd1
Cpt1a
Ppara
Relative mRNA expression (folds of control)
D
Liver
TRH
DAPI
Merged
Enlarged
Ash-NC
Ash-Thrb
200 μm
50 μm
THRβ fluorescence intensity (folds of control, %)
E
F
G
Acetyl-Histone H3 (K9)
Histone H3
Acetyl-Histone H4 (K16)
Histone H4
β-actin
PA
l-THY (nM)
0 0 12 25 50 100
H
IgG
anti-HDAC3
IP: HDAC3
Input
NcoR1
HDAC3
β-actin
I
Relative Cpt1a mRNA expression (folds of control)
si-NC
si-Thrb
J
Relative Ppara mRNA expression (folds of control)
K
THRβ
ns

Supporting Information Figure S5 *l*-THY has a markedly suppression on hepatocyte lipid accumulation.

(A) Quantification for the area of hepatic lipid accumulation was relative to HFD group in HFD-fed C57BL/6J mice treated with ART (20 mg/kg), THI (15 mg/kg) or *l*-THY (1 mg/kg) (n = 6 visual fields each group).

(B) Quantification for the area of hepatic lipid damage was relative to HFD group in HFD-fed C57BL/6J mice treated with ART (20 mg/kg), THI (15 mg/kg) or *l*-THY (1 mg/kg) (n = 6 visual fields each group).

(C) *Scd1*, *Cpt1a* and *Ppara* mRNA levels of HFD-fed C57BL/6J mice treated with ART (20 mg/kg), THI (15 mg/kg) or *l*-THY (1 mg/kg) (n = 6–10 mice each group).

(D) THRβ immunofluorescence staining of liver in Ash-NC or Ash-*Thrb*-injected C57BL/6J mice; quantification for THRβ fluorescence intensity was relative to Ash-NC group (n = 6 visual fields each group).

(E) Quantification for the area of hepatic lipid accumulation was relative to HFD group in Ash-*Thrb*-injected C57BL/6J mice treated with HFD or ART (20 mg/kg) (n = 6 visual fields each group).

(F) Quantification for the area of hepatic lipid damage was relative to HFD group in Ash-*Thrb*-injected C57BL/6J mice treated with HFD or ART (20 mg/kg) (n = 6 visual fields each group).

(G) Immunoblots of Acetyl-Histone H3, Histone H3, Acetyl-Histone H4 and Histone H4 in PA (100 μM)-induced LO2 cells treated with *l*-THY.

(H) Co-IP and western blotting analysis of NcoR1 and HDAC3 in PA (100 μM)-induced LO2 cells treated with *l*-THY.

(I–J) LO2 cells were transfected to express si-NC or si-*Thrb* and treated with PA (100 μM) or *l*-THY (100 nM); *Cpt1a* (I) and *Ppara* (J) mRNA assay were shown (n = 3–4 each group).

(K) LO2 cells were transfected to express si-NC or si-*Thrb* and immunoblots of THRβ were shown.

Statistical comparisons were analyzed by one-way ANOVA analysis followed by Student's t-test. Data are presented as mean ± SD. $^{**}p < 0.01$.

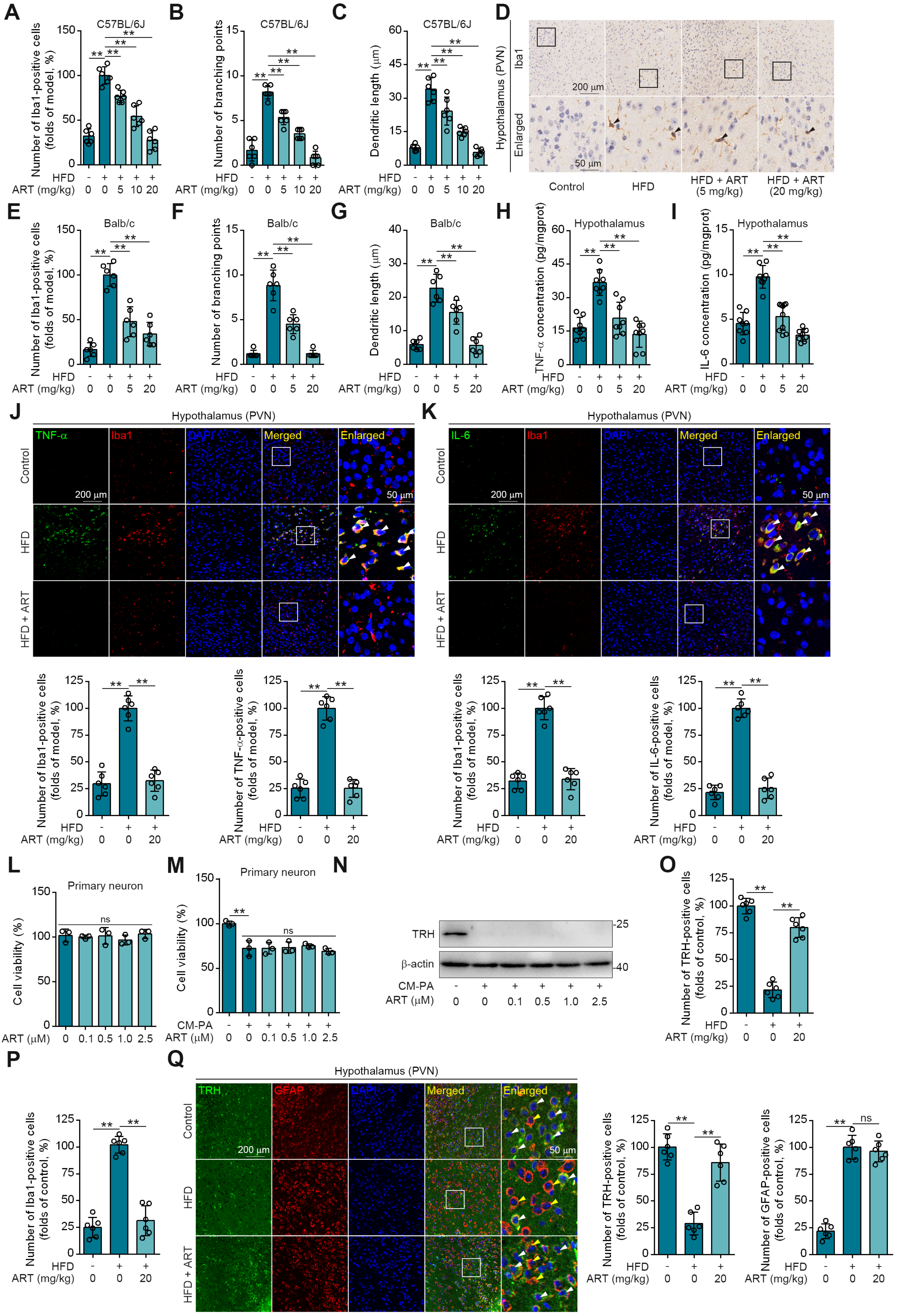
A
C57BL/6J
Number of Iba1-positive cells (folds of model, %)
HFD
ART (mg/kg)
B
Number of branching points
C
Dendritic length (μm)
D
Hypothalamus (PVN)
Iba1
Enlarged
200 μm
50 μm
Control
HFD
HFD + ART (5 mg/kg)
HFD + ART (20 mg/kg)
E
Balb/c
F
G
H
Hypothalamus
TNF-α concentration (pg/mgprot)
I
IL-6 concentration (pg/mgprot)
J
Hypothalamus (PVN)
TNF-α
Iba1
DAPI
Merged
Enlarged
Control
HFD
HFD + ART
Number of TNF-α-positive cells (folds of model, %)
K
IL-6
Number of IL-6-positive cells (folds of model, %)
L
Primary neuron
Cell viability (%)
ns
ART (μM)
M
CM-PA
N
TRH
β-actin
-25
-40
O
Number of TRH-positive cells (folds of control, %)
P
Number of Iba1-positive cells (folds of control, %)
Q
GFAP
Number of GFAP-positive cells (folds of control, %)

Supporting Information Figure S6 ART protects neurons from neuroinflammation in PVN region.

(A) Quantification for the number of Iba1-positive cells was relative to HFD group in HFD-fed C57BL/6J mice treated with ART (n = 6 visual fields each group).

(B–C) Quantification for branching points (B), and dendritic length (C) of HFD-fed C57BL/6J mice treated with ART (n = 6 visual fields each group).

(D) Iba1 immunohistochemical staining of PVN region in HFD-fed Balb/c mice treated with ART.

(E) Quantification for the number of Iba1-positive cells was relative to HFD group in HFD-fed Balb/c mice treated with ART (n = 6 visual fields each group).

(F–G) Quantification for branching points (F), and dendritic length (G) of HFD-fed Balb/c mice treated with ART (n = 6 visual fields each group).

(H–I) TNF-α (H), and IL-6 (I) assay of hypothalamus in HFD-fed Balb/c mice treated with ART (n = 8–10 mice each group).

(J-K) TNF-α (J)/IL-6 (K) and Iba1 immunofluorescence double staining of PVN region in HFD-fed C57BL/6J mice treated with ART; quantification for the number of TNF-α/IL-6 or Iba1 -positive cells was relative to HFD group (n = 6 visual fields each group).

(L) Viability of ART-treated primary neurons in hypothalamus (n = 3 each group).

(M) Viability of CM-PA (100 μM)-induced primary neurons treated with ART in hypothalamus (n = 3 each group).

(N) Immunoblots of TRH from hypothalamus in CM-PA (100 μM)-induced primary neurons treated with ART.

(O) Quantification for the number of TRH-positive cells was relative to control group in HFD-fed C57BL/6J mice treated with ART (n = 6 visual fields each group).

(P) Quantification for the number of Iba1-positive cells was relative to HFD group in HFD-fed C57BL/6J mice treated with ART (n = 6 visual fields each group).

(Q) TRH and GFAP immunofluorescence double staining of PVN region in HFD-fed C57BL/6J mice treated with ART; quantification for the number of TRH-positive cells was relative to control group. Quantification for the number of GFAP-positive cells was relative to HFD group (n = 6 visual fields each group).

Statistical comparisons were analyzed by one-way ANOVA analysis followed by Student's t-test. Data are presented as mean ± SD. $^{**}p < 0.01$.

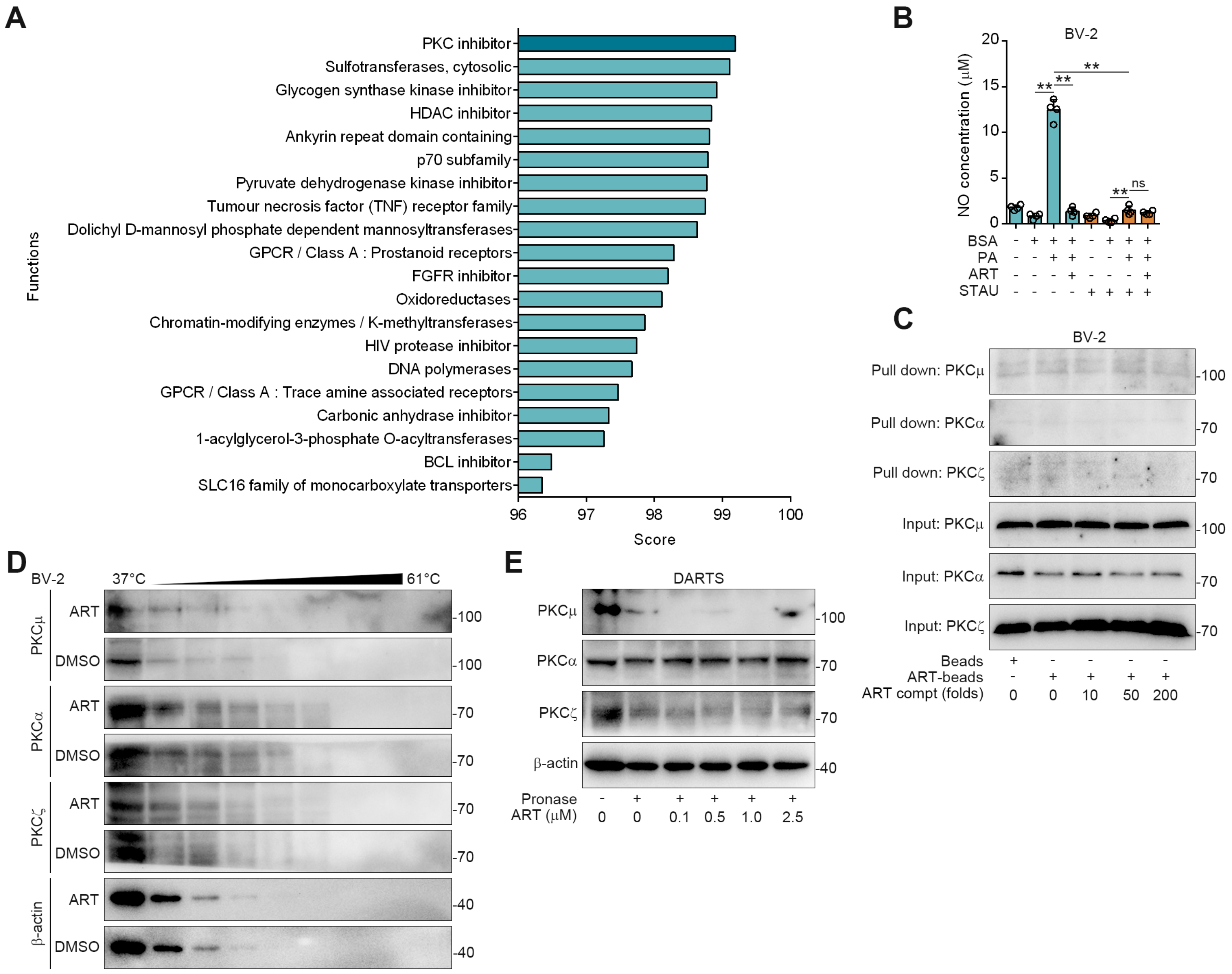

A
PKC inhibitor
Sulfotransferases, cytosolic
Glycogen synthase kinase inhibitor
HDAC inhibitor
Ankyrin repeat domain containing
p70 subfamily
Pyruvate dehydrogenase kinase inhibitor
Tumour necrosis factor (TNF) receptor family
Dolichyl D-mannosyl phosphate dependent mannosyltransferases
GPCR / Class A : Prostanoid receptors
FGFR inhibitor
Oxidoreductases
Chromatin-modifying enzymes / K-methyltransferases
HIV protease inhibitor
DNA polymerases
GPCR / Class A : Trace amine associated receptors
Carbonic anhydrase inhibitor
1-acylglycerol-3-phosphate O-acyltransferases
BCL inhibitor
SLC16 family of monocarboxylate transporters
Functions
96 97 98 99 100
Score
B
BV-2
NO concentration (μM)
20 15 10 5 0
** ** ** ** ns
BSA - + + + - + + +
PA - - + + - - + +
ART - - - + - - - +
STAU - - - - + + + +
C
BV-2
Pull down: PKCμ -100
Pull down: PKCα -70
Pull down: PKCζ -70
Input: PKCμ -100
Input: PKCα -70
Input: PKCζ -70
Beads + - - - -
ART-beads - + + + +
ART compt (folds) 0 0 10 50 200
D
BV-2 37°C 61°C
PKCμ ART -100
DMSO -100
PKCα ART -70
DMSO -70
PKCζ ART -70
DMSO -70
β-actin ART -40
DMSO -40
E
DARTS
PKCμ -100
PKCα -70
PKCζ -70
β-actin -40
Pronase - + + + + +
ART (μM) 0 0 0.1 0.5 1.0 2.5

Supporting Information Figure S7 ART have a selective interaction with PKCδ.

(A) Connectivity Map for predicted functions of ART.

(B) NO assay of PA (100 μM)-induced BV-2 cells treated with ART (2.5 μM) or STAU (1 μM) (n = 4 each group).

(C) BV-2 cell lysates were incubated with ART-biotin followed by pull-down assay by using streptavidin beads; immunoblots of PKCμ, PKCα and PKCζ were shown.

(D) BV-2 cells were treated with DMSO or ART (2.5 μM) and then heated at the indicated temperatures for 3 min; immunoblots of PKCμ, PKCα and PKCζ were shown.

(E) BV-2 cell lysates were incubated with ART and then lysates subjected to pronase digestion; immunoblots of PKCμ, PKCα and PKCζ were shown.

Statistical comparisons were analyzed by one-way ANOVA analysis followed by Student's t-test. Data are presented as mean ± SD. $^{**}p < 0.01$.

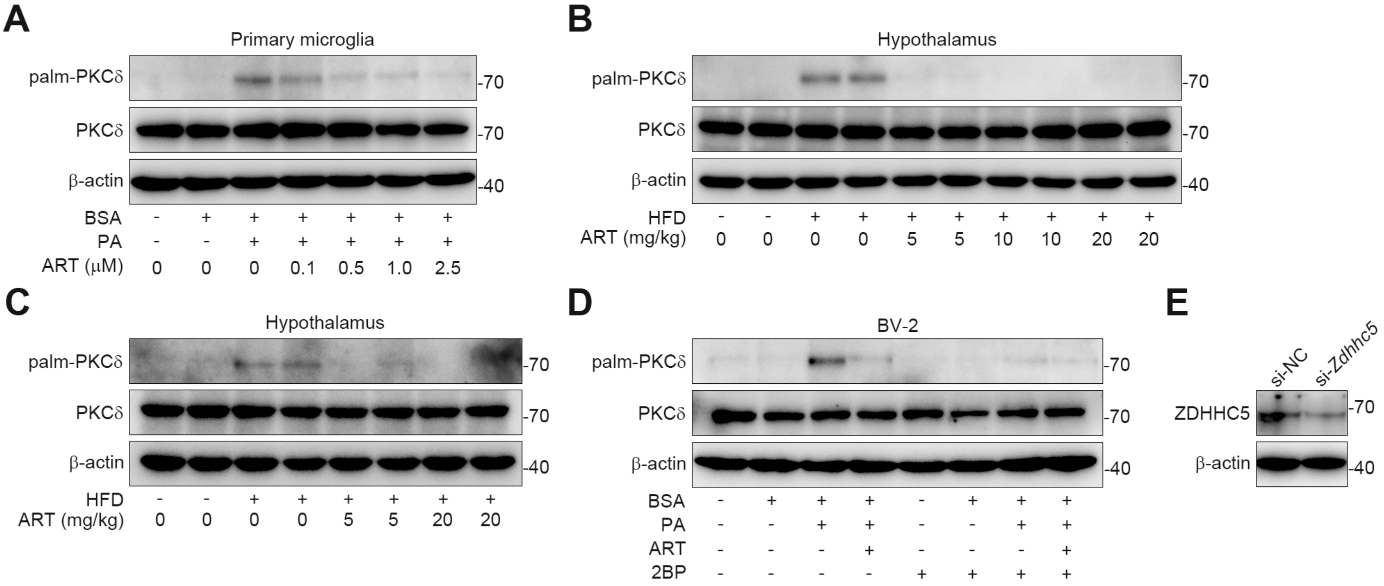
A
Primary microglia
palm-PKCδ
PKCδ
β-actin
-70
-70
-40
BSA - + + + + + +
PA - - + + + + +
ART (μM) 0 0 0 0.1 0.5 1.0 2.5
B
Hypothalamus
palm-PKCδ
PKCδ
β-actin
-70
-70
-40
HFD - - + + + + + + + +
ART (mg/kg) 0 0 0 0 5 5 10 10 20 20
C
Hypothalamus
palm-PKCδ
PKCδ
β-actin
-70
-70
-40
HFD - - + + + + + +
ART (mg/kg) 0 0 0 0 5 5 20 20
D
BV-2
palm-PKCδ
PKCδ
β-actin
-70
-70
-40
BSA - + + + - + + +
PA - - + + - - + +
ART - - - + - - - +
2BP - - - - + + + +
E
si-NC
si-Zdhhc5
ZDHHC5
β-actin
-70
-40

Supporting Information Figure S8 PKCδ palmitoylation was down-regulated significantly by ART.

(A) Immunoblots of palm-PKCδ and PKCδ in PA (100 μM)-induced primary microglia treated with ART (2.5 μM).

(B) Immunoblots of palm-PKCδ and PKCδ hypothalamus in HFD-fed C57BL/6J mice treated with ART.

(C) Immunoblots of palm-PKCδ and PKCδ hypothalamus in HFD-fed Balb/c mice treated with ART.

(D) Immunoblots of palm-PKCδ and PKCδ in PA (100 μM)-induced BV-2 cells treated with ART (2.5 μM) or 2BP (1 μM).

(E) BV-2 cells were transfected to express si-NC or si-*Zdhhc5* and immunoblots of ZDHHC5 were shown.

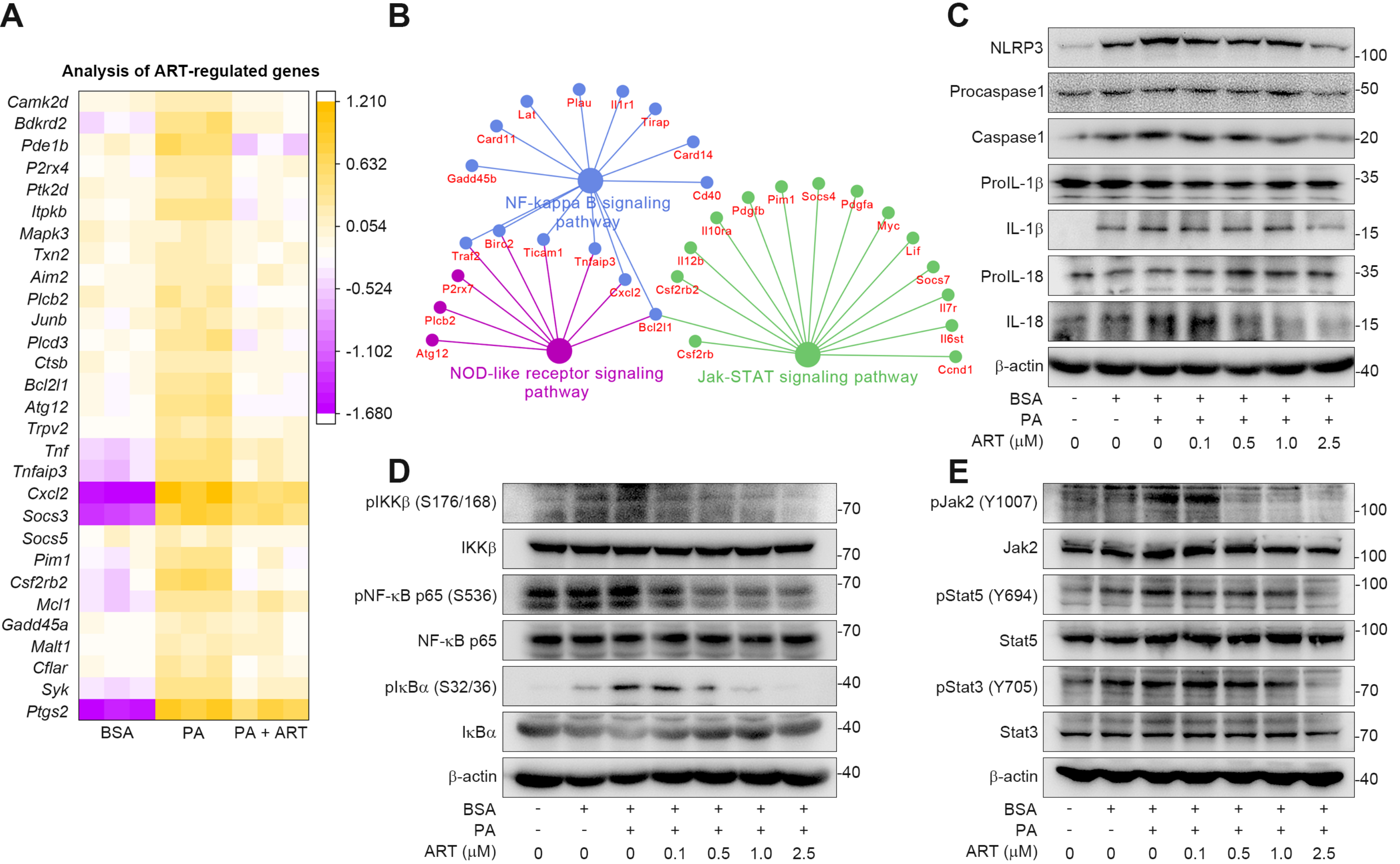
A
Analysis of ART-regulated genes
Camk2d
Bdkrd2
Pde1b
P2rx4
Ptk2d
Itpkb
Mapk3
Txn2
Aim2
Plcb2
Junb
Plcd3
Ctsb
Bcl2l1
Atg12
Trpv2
Tnf
Tnfaip3
Cxcl2
Socs3
Socs5
Pim1
Csf2rb2
Mcl1
Gadd45a
Malt1
Cflar
Syk
Ptgs2
BSA
PA
PA + ART
1.210
0.632
0.054
-0.524
-1.102
-1.680
B
NF-kappa B signaling pathway
NOD-like receptor signaling pathway
Jak-STAT signaling pathway
Lat
Plau
Il1r1
Tirap
Card11
Card14
Gadd45b
Cd40
Traf2
Birc2
Ticam1
Tnfaip3
Cxcl2
Bcl2l1
P2rx7
Plcb2
Atg12
Pdgfb
Pim1
Socs4
Pdgfa
Il10ra
Myc
Lif
Il12b
Csf2rb2
Socs7
Il7r
Il6st
Csf2rb
Ccnd1
C
NLRP3
Procaspase1
Caspase1
ProIL-1β
IL-1β
ProIL-18
IL-18
β-actin
BSA
PA
ART (μM)
D
pIKKβ (S176/168)
IKKβ
pNF-κB p65 (S536)
NF-κB p65
pIκBα (S32/36)
IκBα
β-actin
E
pJak2 (Y1007)
Jak2
pStat5 (Y694)
Stat5
pStat3 (Y705)
Stat3
β-actin
BSA - + + + + + +
PA - - + + + + +
ART (μM) 0 0 0 0.1 0.5 1.0 2.5

Supporting Information Figure S9 ART inhibited PKCδ-related neuroinflammatory signaling pathways.

(A) Gene expressions of NF-κB signaling pathway, Jak-Stat signaling pathway, NOD-like receptor signaling pathway and calcium signaling pathway in PA (100 μM)-induced BV-2 cells treated with ART (2.5 μM).

(B) KEGG analysis of ART-regulated inflammatory pathways.

(C) Immunoblots of NLRP3, caspase1, IL-1β and IL-18 in PA (100 μM)-induced BV-2 cells treated with ART.

(D) Immunoblots of pIKKβ, pNF-κB p65 and pIκBα in PA (100 μM)-induced BV-2 cells treated with ART.

(E) Immunoblots of pJak2, pStat5 and pStat3 in PA (100 μM)-induced BV-2 cells treated with ART.

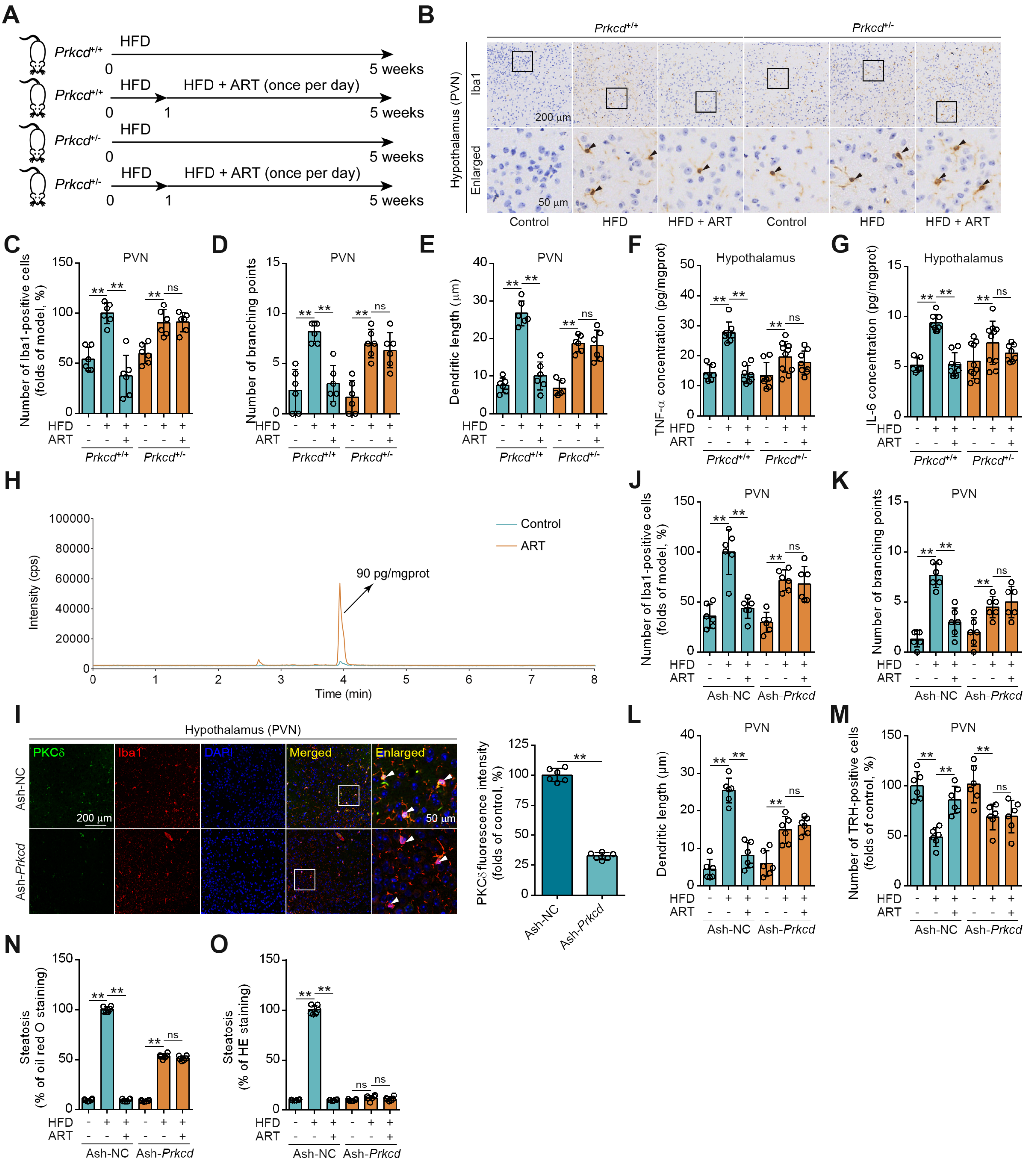

A
Prkcd+/+
HFD
0
5 weeks
Prkcd+/+
HFD
HFD + ART (once per day)
0
1
5 weeks
Prkcd+/-
HFD
0
5 weeks
Prkcd+/-
HFD
HFD + ART (once per day)
0
1
5 weeks
B
Prkcd+/+
Prkcd+/-
Hypothalamus (PVN)
Iba1
Enlarged
200 μm
50 μm
Control
HFD
HFD + ART
Control
HFD
HFD + ART
C
PVN
Number of Iba1-positive cells (folds of model, %)
HFD
ART
Prkcd+/+
Prkcd+/-
D
PVN
Number of branching points
E
PVN
Dendritic length (μm)
F
Hypothalamus
TNF-α concentration (pg/mgprot)
G
Hypothalamus
IL-6 concentration (pg/mgprot)
H
Intensity (cps)
Time (min)
Control
ART
90 pg/mgprot
I
Hypothalamus (PVN)
PKCδ
Iba1
DAPI
Merged
Enlarged
Ash-NC
Ash-Prkcd
200 μm
50 μm
PKCδ fluorescence intensity (folds of control, %)
J
PVN
Number of Iba1-positive cells (folds of model, %)
Ash-NC
Ash-Prkcd
K
PVN
Number of branching points
L
PVN
Dendritic length (μm)
M
PVN
Number of TRH-positive cells (folds of control, %)
N
Steatosis (% of oil red O staining)
O
Steatosis (% of HE staining)
ns
**

Supporting Information Figure S10 Systemic *Prkcd* knockdown reverses ART suppression on lipid metabolism disorder-related neuroinflammation in C57BL/6J mice.

(A) Experimental design of ART treatment in *Prkcd*$^{+/+}$ or *Prkcd*$^{+/-}$ mice fed on HFD.

(B) Iba1 immunohistochemical staining of PVN region in HFD-fed *Prkcd*$^{+/+}$ or *Prkcd*$^{+/-}$ mice treated with ART (20 mg/kg).

(C) Quantification for the number of Iba1-positive cells was relative to HFD group in HFD-fed *Prkcd*$^{+/+}$ or *Prkcd*$^{+/-}$ mice treated with ART (20 mg/kg) (n = 6 visual fields each group).

(D–E) Quantification for branching points (D), and dendritic length (E) of HFD-fed *Prkcd*$^{+/+}$ or *Prkcd*$^{+/-}$ mice treated with ART (20 mg/kg) (n = 6 visual fields each group).

(F–G) TNF-α (F), and IL-6 (G) assay of hypothalamus in HFD-fed *Prkcd*$^{+/+}$ or *Prkcd*$^{+/-}$ mice treated with ART (20 mg/kg) (n = 8–10 mice each group).

(H) UPLC-MS/MS assay of ART in hypothalamus.

(I) PKCδ and Iba1 immunofluorescence double staining of PVN region in Ash-NC or Ash-*Prkcd*-injected C57BL/6J mice; quantification for PKCδ fluorescence intensity in Iba1-positive cells was relative to Ash-NC group (n = 6 visual fields each group).

(J) Quantification for the number of Iba1-positive cells was relative to HFD group in Ash-*Prkcd*-injected C57BL/6J mice treated with HFD or ART (20 mg/kg) (n = 6 visual fields each group).

(K–L) Quantification for branching points (K), and dendritic length (L) of Ash-*Prkcd*-injected C57BL/6J mice treated with HFD or ART (20 mg/kg) (n = 6 visual fields each group).

(M) Quantification for the number of TRH-positive cells was relative to control group in Ash-*Prkcd*-injected C57BL/6J mice treated with HFD or ART (20 mg/kg) (n = 6 visual fields each group).

(N) Quantification for the area of hepatic lipid accumulation was relative to HFD group in Ash-*Prkcd*-injected C57BL/6J mice treated with HFD or ART (20 mg/kg) (n = 6 visual fields each group).

(O) Quantification for the area of hepatic lipid damage was relative to HFD group in Ash-*Prkcd*-injected C57BL/6J mice treated with HFD or ART (20 mg/kg) (n = 6 visual fields each group).

Statistical comparisons were analyzed by one-way ANOVA analysis followed by Student's t-test. Data are presented as mean ± SD. $^{**}p < 0.01$.

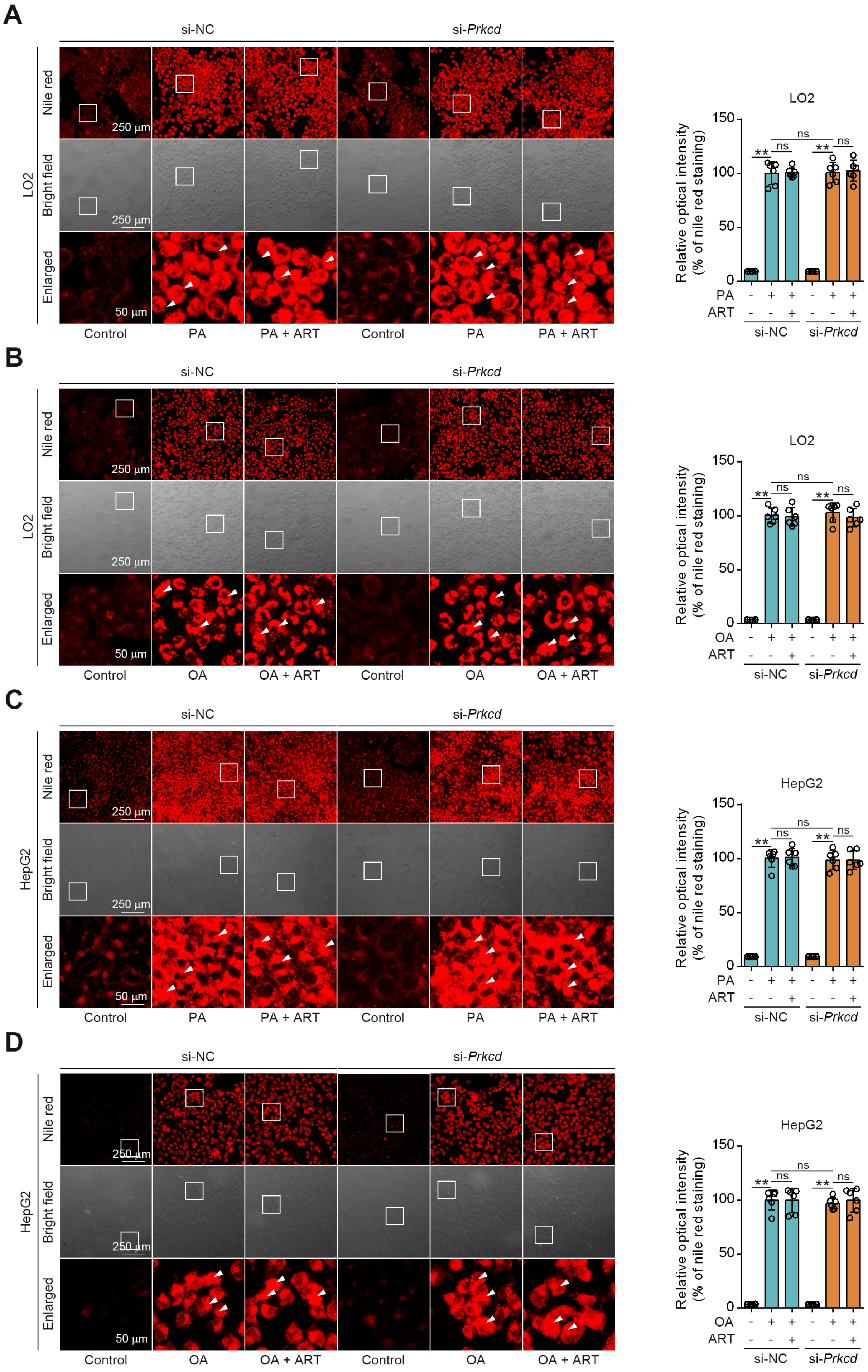

A
si-NC
si-Prkcd
LO2
Nile red
Bright field
Enlarged
250 μm
50 μm
Control
PA
PA + ART
LO2
Relative optical intensity (% of nile red staining)
150
100
50
0
**
ns
ns
ns
PA
ART
B
OA
OA + ART
C
HepG2
D

Supporting Information Figure S11 *Prkcd* knockdown has no effect on hepatocyte lipid accumulation.

(A) LO2 cells were transfected to express si-NC or si-*Prkcd* and treated with PA (100 μM) or ART (2.5 μM); nile red staining of hepatocyte lipid accumulation was shown; quantification for immunofluorescence intensity was relative to PA group (n = 6 visual fields each group).

(B) LO2 cells were transfected to express si-NC or si-*Prkcd* and treated with OA (300 μM) or ART (2.5 μM); nile red staining of hepatocyte lipid accumulation was shown; quantification for immunofluorescence intensity was relative to OA group (n = 6 visual fields each group).

(C) HepG2 cells were transfected to express si-NC or si-*Prkcd* and treated with PA (100 μM) or ART (2.5 μM); nile red staining of hepatocyte lipid accumulation was shown; quantification for immunofluorescence intensity was relative to PA group (n = 6 visual fields each group).

(D) HepG2 cells were transfected to express si-NC or si-*Prkcd* and treated with OA (300 μM) or ART (2.5 μM); nile red staining of hepatocyte lipid accumulation was shown; quantification for immunofluorescence intensity was relative to OA group (n = 6 visual fields each group).

Statistical comparisons were analyzed by one-way ANOVA analysis followed by Student's t-test. Data are presented as mean ± SD. $^{**}p < 0.01$.

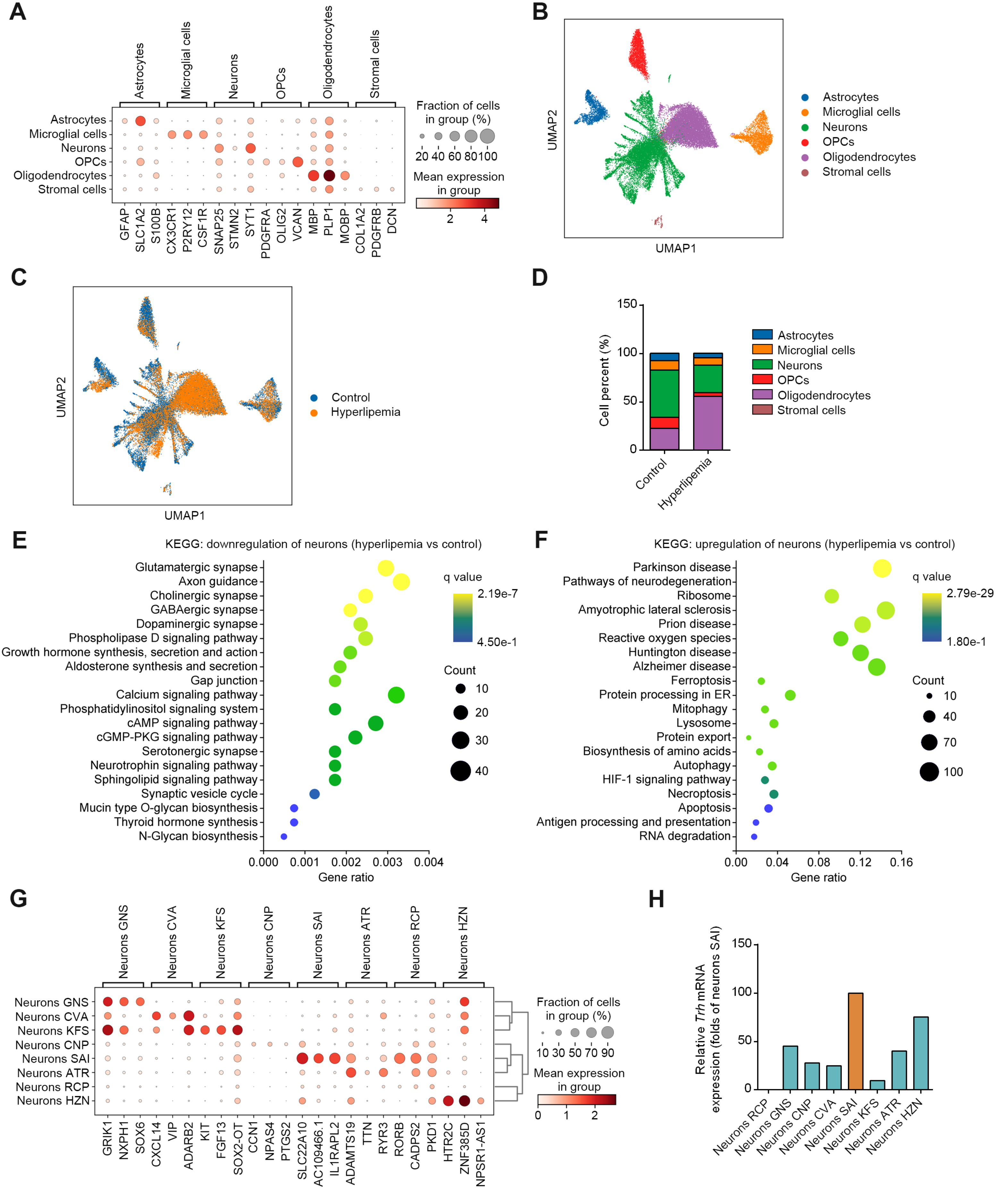

A
Astrocytes
Microglial cells
Neurons
OPCs
Oligodendrocytes
Stromal cells
GFAP SLC1A2 S100B CX3CR1 P2RY12 CSF1R SNAP25 STMN2 SYT1 PDGFRA OLIG2 VCAN MBP PLP1 MOBP COL1A2 PDGFRB DCN
Fraction of cells in group (%)
20 40 60 80 100
Mean expression in group
2 4
B
UMAP2
UMAP1
Astrocytes
Microglial cells
Neurons
OPCs
Oligodendrocytes
Stromal cells
C
UMAP2
UMAP1
Control
Hyperlipemia
D
Cell percent (%)
150
100
50
0
Control
Hyperlipemia
Astrocytes
Microglial cells
Neurons
OPCs
Oligodendrocytes
Stromal cells
E
KEGG: downregulation of neurons (hyperlipemia vs control)
Glutamatergic synapse
Axon guidance
Cholinergic synapse
GABAergic synapse
Dopaminergic synapse
Phospholipase D signaling pathway
Growth hormone synthesis, secretion and action
Aldosterone synthesis and secretion
Gap junction
Calcium signaling pathway
Phosphatidylinositol signaling system
cAMP signaling pathway
cGMP-PKG signaling pathway
Serotonergic synapse
Neurotrophin signaling pathway
Sphingolipid signaling pathway
Synaptic vesicle cycle
Mucin type O-glycan biosynthesis
Thyroid hormone synthesis
N-Glycan biosynthesis
0.000 0.001 0.002 0.003 0.004
Gene ratio
q value
2.19e-7
4.50e-1
Count
10
20
30
40
F
KEGG: upregulation of neurons (hyperlipemia vs control)
Parkinson disease
Pathways of neurodegeneration
Ribosome
Amyotrophic lateral sclerosis
Prion disease
Reactive oxygen species
Huntington disease
Alzheimer disease
Ferroptosis
Protein processing in ER
Mitophagy
Lysosome
Protein export
Biosynthesis of amino acids
Autophagy
HIF-1 signaling pathway
Necroptosis
Apoptosis
Antigen processing and presentation
RNA degradation
0.00 0.04 0.08 0.12 0.16
Gene ratio
q value
2.79e-29
1.80e-1
Count
10
40
70
100
G
Neurons GNS
Neurons CVA
Neurons KFS
Neurons CNP
Neurons SAI
Neurons ATR
Neurons RCP
Neurons HZN
GRIK1 NXPH1 SOX6 CXCL14 VIP ADARB2 KIT FGF13 SOX2-OT CCN1 NPAS4 PTGS2 SLC22A10 AC109466.1 IL1RAPL2 ADAMTS19 TTN RYR3 RORB CADPS2 PKD1 HTR2C ZNF385D NPSR1-AS1
Fraction of cells in group (%)
10 30 50 70 90
Mean expression in group
0 1 2
H
Relative *Trh* mRNA expression (folds of neurons SAI)
150
100
50
0
Neurons RCP
Neurons GNS
Neurons CNP
Neurons CVA
Neurons SAI
Neurons KFS
Neurons ATR
Neurons HZN

Supporting Information Figure S12 PKCδ expression is upregulated in fatty brain and correlates with hyperlipemia progression.

(A) Marker gene dot plot of single-nuclei transcriptomic data of brain tissue.

(B) UMAP plot of single-nuclei transcriptomic data from brain tissue of all patient samples.

(C) Comparison of UMAP plot between control and hyperlipemia brain tissue.

(D) Cell percentage of single-nuclei transcriptomic data of brain tissue.

(E) KEGG analysis of top 20 down-regulated pathways of neurons (hyperlipemia vs control).

(F) KEGG analysis of top 20 up-regulated pathways of neurons (hyperlipemia vs control).

(G) Top marker gene dot plot of single-nuclei transcriptomic data in neurons.

(H) *Trh* expressions in different type of control neurons.

Supporting Information Reaction

Synthesis of biotin-ART

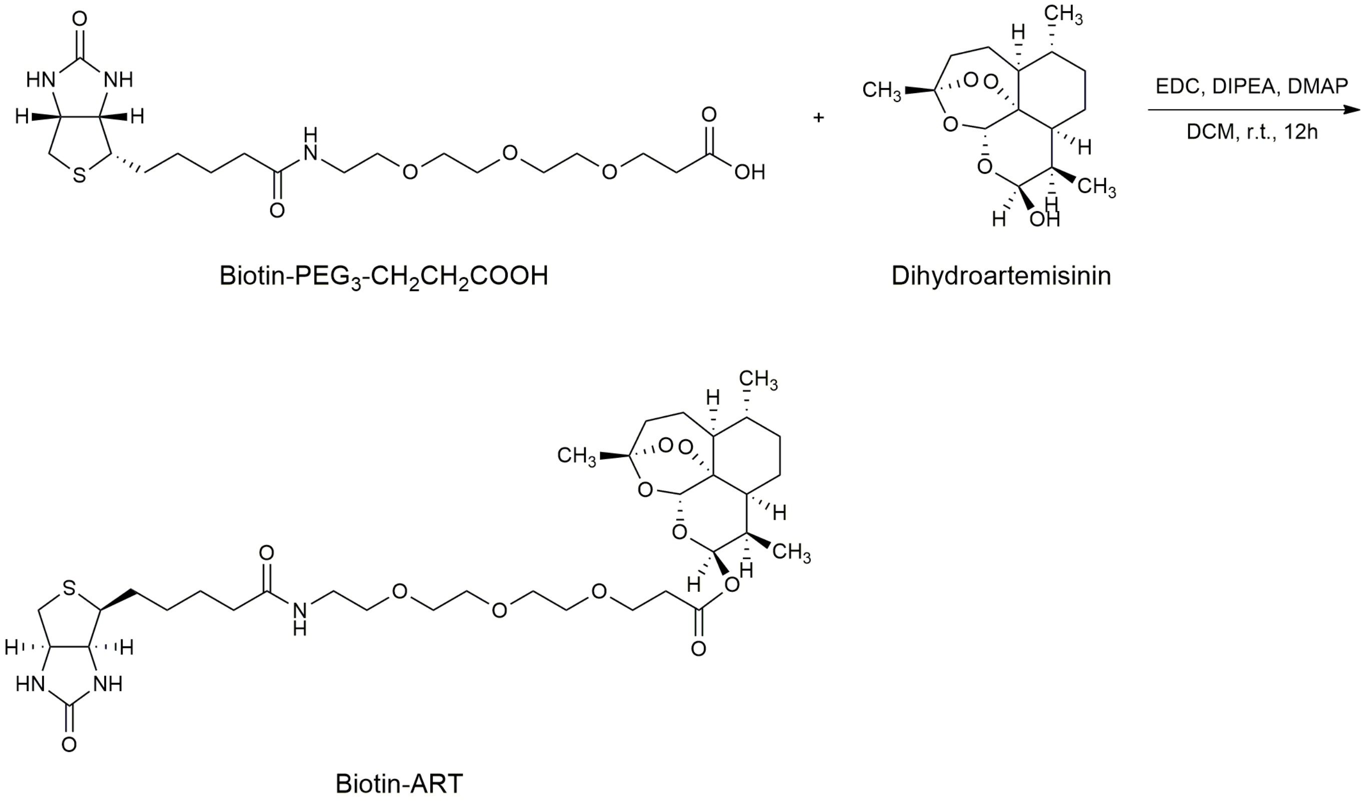

Synthesis of biotin-ART

To a suspension of Biotin-PEG$_3$-CH$_2$CH$_2$COOH (22.38 mg, 50 μmol) and dihydroartemisinin (14.22 mg, 50 μmol) in DCM (5 mL) were added EDC (11.5 mg, 60 μmol), DIPEA (20 μL) and DMAP (0.61 mg, 5 μmol). The resulting mixture was kept at room temperature with shaking for 24 h. The residue was purified by column chromatography on silica gel (0-10% MeOH in DCM) to give biotin-artemether as an off yellow solid (20.2 mg, 57%). ^{1}H NMR (500 MHz, CD$_3$OD) δ 5.73 (d, J = 9.5 Hz 1H), 5.49 (s, 1H), 4.44 (dd, J = 7.5, 5.0 Hz, 1H), 4.26 (dd, J = 7.5, 4.0 Hz, 1H), 3.73 (t, J = 5.5 Hz, 2H), 3.49 (t, J = 4.9 Hz, 2H), 3.32 (d, J = 5.0 Hz 2H), 3.26 (s, 1H), 3.20-3.13 (m, 1H), 2.88 (dd, J = 7.5, 5.0 Hz, 1H), 2.71-2.60 (m, 3H), 2.42 (dd, J = 13.5, 3.0 Hz, 1H), 2.17 (t, J = 7.2 Hz, 2H), 2.00 (d, J = 14.5 Hz 1H), 1.92-1.83 (m, 1H), 1.79-1.50 (m, 10H), 1.48-1.34 (m, 7H), 1.31 (s, 3H), 1.28-1.20 (m, 3H), 0.99 (d, J = 13.0 Hz, 1H), 0.92 (d, J = 6.5 Hz, 4H), 0.83 (d, J = 7.0 Hz, 4H). ^{13}C NMR (125 MHz, CD$_3$OD) δ 176.07, 171.95, 166.04, 105.62, 93.57, 92.87, 81.37, 71.60, 71.50, 71.41, 71.24, 70.57, 67.37, 63.34, 61.60, 57.00, 52.97, 46.56, 41.06, 40.37, 38.25, 37.28, 36.74, 36.05, 35.30, 33.09, 29.78, 29.50, 26.85, 25.93, 25.78, 22.86, 20.60, 12.47. (+)-ESIMS m/z 736.1 [M + Na]$^+$.

Supplemental Table 1

Protein name	Intensity L1	Intensity L2	Intensity L3	Intensity H1	Intensity H2	Intensity H3
Tubb2a	81161000	60859000	70025000	0	0	0
Sec61b	49479000	53750000	58225000	0	0	0
Timm8a1	25353000	25638000	26238000	0	0	0
Prkab1	21091000	20570000	17124000	0	0	0
Ndufb9	16707000	16389000	25585000	0	0	0
Atp5e	16378000	12869000	17238000	0	0	0
Ufd1	19373000	12427000	12138000	0	0	0
Pnkp	11404000	12491000	11651000	0	0	0
H-2 class I h	10528000	10608000	11680000	0	0	0
Dnajc5	10406000	7292800	11156000	0	0	0
Mospd2	9846100	6426600	7698500	0	0	0
Coro1b	6518300	10501000	6212700	0	0	0
Mrps18b	7170900	7044200	7468700	0	0	0
Prkcd	5214780	6874123	8660000	0	0	0
Sqle	3695200	4876500	5410100	0	0	0
Glmp	40898000	34722000	28705000	7640300	6015800	5054000
Alyref	52398000	49150000	48684000	10112000	12274000	14980000
Nucb2	27887000	39706000	18306000	7105500	14941000	3764100
Cops2	29792000	47606000	36208000	5408900	19607000	12123000
Birc6	20345000	24446000	25479000	4326500	5446600	13510000
Edf1	58764000	50689000	35605000	15771000	21912000	15597000
Atp6v1h	69957000	69448000	94882000	7369800	32464000	51180000
Sord	134820000	76771000	170830000	40155000	12539000	96929000
Sugt1	61114000	75379000	89151000	25216000	30500000	32880000
Rab2a	29436000	48702000	47372000	3273500	19415000	27536000
Cyfip1	29588000	61300000	41087000	3179000	26377000	23984000
Mrpl37	22194000	18446000	22787000	7287300	6580200	12008000
Napa	99359000	82889000	89833000	50014000	16877000	45183000
Pdhb	392350000	402690000	387880000	140060000	145830000	202870000
Git1	27310000	29979000	23756000	13181000	16354000	4567100
Snrpa1	59250000	60949000	58354000	28199000	24370000	22982000
Alg5	41803000	46914000	36618000	20723000	19203000	13845000
Serpinb1a	451690000	335520000	443550000	181970000	190560000	157960000
Agps	127980000	87083000	115510000	69102000	39274000	40190000
Sptlc2	23426000	16652000	20183000	13023000	7111000	7451200
Dab2	107330000	89047000	87481000	59616000	50342000	23100000
Ppp2r5c	14436000	13545000	20293000	6999700	4054000	11688000
Eif2a	91622000	106750000	131150000	52305000	47874000	56042000
Cers2	84487000	89300000	93364000	37542000	51765000	38750000
JPT1	116300000	123270000	130680000	57115000	62247000	61149000
Ncf4	12892000	16084000	9696000	3719100	11782000	3434400
Ubxn1	24100000	55261000	34465000	4664000	33130000	19133000
Chordc1	33885000	82817000	74324000	7530100	44566000	43574000
Sra1	12063000	11757000	8625400	6661900	5773200	3945500
Dlst	288590000	395390000	361850000	189930000	167240000	170840000
Eif6	261070000	224260000	332980000	131950000	128590000	152660000
Nt5c2	203970000	163340000	181210000	95233000	76247000	106750000
Plekho2	15120000	27789000	29561000	2114700	19590000	15402000
Arpc5	64502000	126750000	100650000	14684000	74322000	60755000
Mvd	171930000	153650000	164710000	69810000	69686000	112940000
Mrps27	29346000	29823000	33591000	17098000	11891000	18983000
Fubp1	102640000	100290000	122710000	53602000	53141000	61880000
Lpl	21020000	23890000	17104000	9136200	13340000	9775500
Wipf1	52141000	39032000	40369000	30788000	15360000	22288000
Hnrnpa3	126460000	108450000	150470000	87786000	33857000	82604000
Adh5	528750000	522640000	382950000	271670000	289750000	204160000
Ctsl	43719000	21944000	47612000	25901000	11117000	23501000
Anp32b	226960000	187670000	231070000	156070000	87578000	101920000

Psme3	135600000	170110000	161300000	53544000	87989000	111170000
Acot2	12627000	20479000	16032000	6624900	10800000	9398600
p53	274430000	321350000	392770000	130720000	193870000	218960000
Arpc3	66567000	91535000	80981000	34287000	53658000	43555000
Atp6v0d1	217720000	205890000	185100000	108630000	112770000	113990000
Xpnpep1	86538000	125730000	78706000	52576000	64081000	44640000
Aldh9a1	98616000	121190000	92390000	59040000	78668000	37012000
Ctsd	436560000	345670000	443510000	231450000	204960000	251760000
Pnp	197540000	183820000	229440000	76939000	93145000	178060000
Pgpep1	9062900	12715000	10479000	6104200	7274600	5116100
Ap2a1	6807100	8011800	8358800	4044600	3869400	5565100
Pdcl3	29284000	54952000	38565000	16089000	31894000	23803000
Gstm1	285940000	280330000	366010000	174360000	174400000	197130000
Nubp1	31550000	19554000	30593000	21702000	5218400	21325000
Gcn1	404180000	401670000	384420000	252980000	209810000	243350000
Hadha	233440000	227950000	415410000	120460000	139620000	260350000
Mapre1	241600000	238560000	288150000	150980000	130980000	175050000
Api5	126780000	142690000	133740000	57543000	78325000	104380000
Arpc4	190530000	243980000	136590000	132520000	138400000	71035000
Actr2	239590000	246050000	273100000	139770000	164280000	151120000
Hexb	9710700	10878000	6631700	5409100	6538200	4415800
Rtn4	535630000	529230000	597020000	284160000	335610000	379550000
Strap	261250000	271880000	288450000	166120000	171930000	156320000
Ndufb10	17945000	16263000	16183000	12063000	11184000	7089700
Fkbp11	85000000	132550000	177610000	52439000	82248000	104050000
Mt2	15987000	19032000	21503000	11298000	10849000	12144000
Rab14	231150000	315050000	219130000	146060000	189760000	135100000
Serpinb6	571580000	492120000	502550000	351200000	305530000	307070000
Gmps	261140000	286540000	208210000	156830000	176840000	131610000
Smndc1	29957000	46926000	22843000	15438000	30192000	15756000
Dbnl	25926000	44135000	44922000	16965000	28476000	25489000
Mthfd1	461850000	533370000	520570000	290180000	332580000	312530000
Cyba	59855000	66389000	79547000	40405000	43033000	44257000
Stx7	7854600	8816100	8860500	4664000	5123400	6167000
Ppp1r2	19909000	20022000	18754000	11811000	13271000	11783000
Tpd52	26020000	39478000	32944000	18626000	26139000	17473000
Acot7	235860000	258760000	223190000	141330000	180660000	132300000
Dctn1	12622000	19398000	17428000	7046300	12158000	12158000
Vapa	222690000	173880000	279950000	133340000	105990000	191160000
Hk1	155740000	169880000	171410000	76996000	123970000	115650000
Chchd4	40412000	49466000	53928000	20987000	32416000	38317000
Dync1li1	139620000	188390000	211110000	97119000	118930000	128830000
Acsl4	201090000	252220000	264750000	127260000	168860000	163370000
Gtf2f1	49117000	68909000	89171000	15204000	45879000	72637000
Psmg1	71367000	63097000	97010000	51012000	39644000	59519000
Slc25a13	140230000	121840000	130160000	86848000	90445000	77624000
Lonp1	589230000	550300000	676170000	370120000	352580000	458720000
Pak2	294070000	285260000	347590000	185640000	168040000	250250000
Ddx1	187120000	180480000	170940000	112850000	127830000	110710000
Romo1	12524000	14422000	17283000	9566100	8637800	10706000
Prdx2	542580000	496740000	502060000	325580000	338060000	344490000
Pdia4	225420000	202890000	247280000	126480000	161010000	154530000
Ipo9	33688000	31226000	33830000	17669000	20344000	26728000
Anxa3	376460000	440820000	435330000	257730000	305850000	261250000
Mdh1	824710000	648280000	862730000	515000000	489330000	534360000
Emc1	39326000	54585000	43840000	27308000	37031000	26460000
Ube2v2	143880000	169900000	251310000	81137000	82376000	209220000
Aars	829620000	941440000	996080000	557110000	590100000	687290000
Tagln2	384720000	314060000	392460000	220530000	206260000	297410000
Ppp2ca	188440000	216750000	177810000	135210000	142720000	110910000

Supplemental Table 2

Protein name	Intensity L1	Intensity L2	Intensity L3	Intensity H1	Intensity H2	Intensity H3
Timm8a1	25353000	25638000	26238000	0	0	0
Prkab1	21091000	20570000	17124000	0	0	0
Ndufb9	16707000	16389000	25585000	0	0	0
Atp5e	16378000	12869000	17238000	0	0	0
Dnajc5	10406000	7292800	11156000	0	0	0
Prkcd	5214780	6874123	8660000	0	0	0
Atp6v1h	69957000	69448000	94882000	7369800	32464000	51180000
Rab2a	29436000	48702000	47372000	3273500	19415000	27536000
Alg5	41803000	46914000	36618000	20723000	19203000	13845000
Serpinb1a	451690000	335520000	443550000	181970000	190560000	157960000
Eif2a	91622000	106750000	131150000	52305000	47874000	56042000
Cers2	84487000	89300000	93364000	37542000	51765000	38750000
Atp6v0d1	217720000	205890000	185100000	108630000	112770000	113990000
Aldh9a1	98616000	121190000	92390000	59040000	78668000	37012000
Ndufb10	17945000	16263000	16183000	12063000	11184000	7089700
Rab14	231150000	315050000	219130000	146060000	189760000	135100000
Serpinb6	571580000	492120000	502550000	351200000	305530000	307070000
Acsl4	201090000	252220000	264750000	127260000	168860000	163370000
Gtf2f1	49117000	68909000	89171000	15204000	45879000	72637000
Slc25a13	140230000	121840000	130160000	86848000	90445000	77624000

Supplemental Table 3

Protein name	Sample 1	Sample 2	Sample 3	Sample 4	Sample 5
Pygb	49385692.42	95619903.91	107839644.2	80020369.29	125919166.9
Sdha	88913675.21	69750710.3	88136907.64	80041799.1	101252710.7
Sucla2	99424174.89	70275193.6	69293535.24	125022729.8	73895182.76
Gpm6a	29064080.72	69825155.91	79722223.67	40952486.53	71485128.21
Aldh5a1	52261211.53	55772387.72	66189923.47	43100131.09	63736670.03
Dlat	67859755.5	56588247.86	58950522.96	90832689.94	63122702.91
Sirt2	78910259.08	24630319.49	50487229.21	69155428.83	46653191.9
Oxct1	56186978.02	41499036.41	35044159.72	68237610.75	41719355.96
Slc3a2	22467891.43	32511978.09	48242052.57	27673284.36	41193978.88
Auh	6982374.961	29632660.6	26586550.59	9152384.188	35007540.41
Pygm	34207463.99	24512809.46	20962987.38	58991570.82	32857379.3
Acat1	99348219.63	37295564.08	43189583.44	134283950.5	29500105.71
Pafah1b2	4942921	34436528.31	21261408.44	829725.1875	28150879.75
Ddah1	18621770.83	22046594.93	27322401.09	27487907.3	25531632.18
Mog	29436847.5	10636250.35	23752102.46	30768528.25	22913156.06
Sdhb	31572342.33	19056865.55	22231366.18	35419987.7	22811441.98
Aldh6a1	10570067.33	22062681.58	23977395.71	6375891.43	21160255.32
Dlst	13360058.06	22417664.38	13806125.82	16733336.53	20708220.28
Pcmt1	9074597.258	33207868.17	31030963.98	9670296.91	20091793.82
Rpn1	6834917.965	12958432.19	21067564.79	8242164.525	17750661.1
Hmgcl	2555407.379	11776305.25	15912161.19	4017490.75	15966289.63
Sv2a	47705931.56	6207645.82	10043804.22	55732142.54	15871049.92
Cd200	427103.2188	7294971.063	17630677.25	286270.4688	15231022.58
Suclg1	31505974.25	6223467.779	15206614.8	19428885.36	14609522.34
Pafah1b1	24036418.73	10252951.68	11875813.16	36413235.77	14563131.58
Acat2	9285028.887	7867176.445	10143511	12316772.28	13220744.84
Adss2	4728163.078	9307203.125	10983177.21	8352892.625	11354260.19
Clic4	11440551.23	6660981.5	5415118.129	15096635.52	8925991.258
Fahd2	5016937.48	7044644.992	8625009.492	6390363.625	8890977.922
Mag	28479865.4	4204210.109	12797737.46	16613902.86	8890043.766
Gsk3a	7419804.822	6529605.943	9128232.281	9312924.121	8081490.535
Selenbp1	2804854.895	9172753.699	7616272.945	3197946.078	7610319.094
Lamp1	1773341.125	7712995	6469682	2409671.5	7326152.5
Thy1	101883528.4	6016644.938	19388476.81	23203559.25	7263158.156
Adsl	3516108.516	6796181.566	5740855.281	5367449.281	6256325.414
Ddah2	751098.6875	6792150.5	715197.4063	8059361.313	5951671.75
Rpn2	4534236.719	4140029.115	6523302.711	6155686.766	5784859.828
Pafah1b3	3594234.406	4690283.012	5611666.023	3952273.688	5727644.262
Acss1	5660888.805	2525881.305	5952394.313	1472746.789	5429265.156
Gstz1	3749378.688	4559523.703	1674658.375	3470367.438	5424931.098
Pdk3	1819742.063	4629148.875	4998007.852	2397421.313	5263957.844
Ppme1	16481875.66	2160858.359	2848687.188	17252140.38	5134307.828
Ahsg	2687533.125	5059390.938	3597610.167	4635557.688	5004129.906
Gsk3b	9536748.75	3916796.428	3565403.234	10952670.69	4747203.064
Adss1	1419005.758	4504149.266	5498601.744	3275125.531	4476788.672
Mat2b	1457887	3517801.82	3781386.578	2599475.781	4247010.828
Aldh9a1	4011949.5	2484209.297	5708943.34	5993614.191	4246161.938
Ddost	1445116.844	3234262.156	5717960.781	2258006.547	4126013.047
Mccc1	1430486.07	1814480.188	3903434.213	2154825.992	4050260.992
Ogt	2867360.631	5441108.594	5236631.295	2657614.52	4011481.859
Hdac2	537192.4375	3305613.719	3038210.672	668835.5313	3552021.922
Prmt1	4756810.137	2817739.379	1835342.828	6193501.758	3414816.625
Pdk2	996320.1875	3856788.563	2716764.896	3055442.09	3341868.969
Uggt1	2222111.085	3222941.961	2113942.258	2673585.055	3172466.438
Mmut	3050510.313	3604960.602	4700534.582	3633070.984	3081492.885
Gltp	12728755.69	2117035.287	2419146.289	19774385.69	2775091.627

Sdhc	1193626.625	2374450.188	2572373.125	2803110.25	2639332.75
Atat1	315155	2222842.531	2513350.875	380954.9063	2538055.719
Sv2b	12994576.72	1049082.152	2670012.35	13555229.97	2500402.082
Pgm3	757752.875	2481390.32	2672607	1163085.453	2276392.668
Prmt5	3450405.094	1437754.541	2030886.813	3906715.938	2268811.5
Naa15	1878412.844	1638688.598	1985603.875	2117149.75	2052382.836
Gpm6b	17915926.75	1891214.063	1311766.998	7788973.656	2006896.859
Nat8l	1614362	1550395.703	2041036.313	1798524.875	1905019.336
Acaca	2992994.283	1453595.393	1176949.766	3406194.027	1895998.516
Comt	1234576.219	1948037.266	1344640.75	1441945.148	1863871.742
Ass1	18593412.75	1338311.516	2324812.922	12472854.34	1772145.09
Fah	346439.7969	1314180.844	1395761	869770.0703	1713126.188
Hmgcs1	1053768.152	1474482.828	1293101.484	3159040.047	1688769.016
Mtap	183204.9531	299962.9688	407443.125	1733680.406	1665396
Metap2	2288906.375	2345240.344	1709473.656	795959.125	1590156.688
Comtd1	6969030.75	1118158.031	1264545.5	6689501.344	1586741.75
Nt5c3b	5856064	1536725.031	1164070.938	5818491.5	1583926.031
Hdac11	1235577.906	1356628.906	1459313.969	1677757	1569796.063
Cd47	7313830.438	1086845.133	1985703.707	9156090.375	1560665.254
Hnmt	2104443.75	1259242.793	1725834.678	2828432.031	1439237
Mccc2	4758476.938	2317724.211	2590085.563	5363727.266	1330806.672
Uap1l1	565534.875	1123674.152	1575728.102	814735.707	1316560.145
Sv2c	1908962.656	1203413.875	888470.25	3437531.75	1285244
Naga	683522.0625	1007719.891	1270459.984	936150.0547	1249032.188
Mars1	2662934.875	2706284.332	1638462.551	3891042.625	1107593.234
Npc2	1163041.375	632788.375	628476.5	1427243.875	1081271.875
Mcam	359348.1094	1166366.016	476741.9063	1043339.406	1066736.094
Mcam	359348.1094	1166366.016	476741.9063	1043339.406	1066736.094
Stt3b	536852.5	760707.0391	158657.625	571918.0625	1035548.203
Rnmt	2850885.633	901539.3125	221295.8125	3561594.367	921791.0313
Tpbg	203139.75	639917.0117	879874.75	349669.875	849446.8262
Naa50	1053559.75	313830.5	486798.6875	202388	759344.125
Naa25	211915.1406	468410.9609	563152.4844	259973.0625	751500.1563
Suclg2	4288580.887	350558.6563	281693.625	5356665.531	724778.0156
Hdac6	2392175	749208.0313	660149.7813	872484.75	722951.9063
Mat2a	5763655.875	903541.0938	581945.6211	8460926.594	640448.4297
Carm1	140975.2031	354415.125	759117.375	259389.5938	623727.6875
Setd7	3496469.938	667732.1719	719564.1563	6242867.375	623553.8047
Asl	2104376.381	411222.6445	681058.7695	942168.3125	564999.6875
Pla2g7	401278.3438	617460.4531	196687.8125	841632.7031	563069.1875
Pcif1	85999.89063	903312.8984	212738.793	601700.25	507206.4375
Habp4	480016.9375	194057.0156	473887.9375	123809.3281	497281.2813
Pdk1	620330.5195	340278.6406	485768.8594	616966.3125	481828.8203
Tpmt	1083357.719	584222.5781	341140.4063	577498.125	468828.7344
Pdp1	24492.25781	673075.7188	1324997.188	190483.9844	457965.4063
Lamp5	404334.375	405885.375	315996.625	96484.65625	447608.8125
Crat	811588.1621	837143.2031	290874.5625	923078.9063	443094.6172
Zdhhc5	71515.75	320639.5625	410283.3438	169583.2969	410499.2188
Hdac5	226213.125	173662.8594	193065.3594	328156.875	383018.8438
Ngly1	74162.10938	550342.9375	412446.5	28181.11328	368762.25
Stt3a	1233862.25	112654.75	173070.0625	1032542.813	336920.75
Sap25	682989.125	142398.75	223618.4063	860398.3125	325389.5313
Pomgnt2	213790.2656	234722.8047	179281.5781	171657.7969	138747.9063
Coq3	231499.6719	43746.24609	67237.72656	176290.125	88456.61719
Prmt3	281272.625	45138.96875	26055.77539	378035.125	57422.79688
Adprs	380349.4375	36794.37891	44339.8125	920559.8594	40667.01563

Supplemental Table 4

Primers

Relating to Star Methods Section Quantitative RT-PCR

Scd1 (mouse)	Forward	CCGGGAGAATATCCTGGTTT
	Reverse	CGGTACTCACTGGCAGAGT
Cpt1a (mouse)	Forward	AGTGGCCTCACAGACTCCAG
	Reverse	GCCCATGTTGTACAGCTTCC
Ppara (mouse)	Forward	CAGGTTTGCGTAGAAGAGC
	Reverse	CAGGTTGACTGGACGGA
Gapdh (mouse)	Forward	GGTGAAGGTCGGTGTGAACG
	Reverse	CTCGCTCCTGGAAGATGGTG
Cpt1a (human)	Forward	CTACACGGCCGATGTTACGA
	Reverse	AGGAGTGTTCAGCGTTGAGG
Ppara (human)	Forward	CTGTCGGGATGTCACACAAC
	Reverse	CGGGCTTTGACCTTGTTCAT
Gapdh (human)	Forward	GAAGCTCACTGGCATGGCCTTC
	Reverse	GACCACCTGGTGCTCAGTGTAG

Supplemental Table 5

UPLC-MS/MS

Relating to Star Methods Section Transport Experiments

Oleic acid (OA)	Declustering potential (DP)	-100
	Collision energy (CE)	-10
Palmitic acid (PA)	Declustering potential (DP)	-100
	Collision energy (CE)	-10
Stearic acid (SA)	Declustering potential (DP)	-100
	Collision energy (CE)	-10
Cholesterol (CHO)	Declustering potential (DP)	100
	Collision energy (CE)	10
Artemether (ART)	Declustering potential (DP)	15
	Collision energy (CE)	18
Artesunate	Declustering potential (DP)	100
	Collision energy (CE)	18
